# Cat: Empirical modelling of *Felis catus* population dynamics in the UK

**DOI:** 10.1371/journal.pone.0287841

**Published:** 2023-07-12

**Authors:** Jenni McDonald, Lauren Finka, Rae Foreman-Worsley, Elizabeth Skillings, Dave Hodgson

**Affiliations:** 1 Feline Welfare Directorate, Cats Protection, National Cat Centre, Haywards Heath, United Kingdom; 2 Bristol Veterinary School, University of Bristol, Bristol, United Kingdom; 3 Centre for Ecology and Conservation, University of Exeter, Cornwall, United Kingdom; National Veterinary Research Institute (NVRI), NIGERIA

## Abstract

Domestic cats are popular companion animals, however not all live in human homes and many cats live within shelters or as free-roaming, unowned- feral or stray cats. Cats can transition between these subpopulations, but the influence of this connectivity on overall population dynamics, and the effectiveness of management interventions, remain poorly understood. We developed a UK-focused multistate Matrix Population Model (MPM), combining multiple life history parameters into an integrated model of cat demography and population dynamics. The model characterises cats according to their age, subpopulation and reproductive status, resulting in a 28-state model. We account for density-dependence, seasonality and uncertainty in our modelled projections. Through simulations, we examine the model by testing the effect of different female owned-cat neutering scenarios over a 10-year projection timespan. We also use the model to identify the vital rates to which total population growth is most sensitive. The current model framework demonstrates that increased prevalence of neutering within the owned cat subpopulation influences the population dynamics of all subpopulations. Further simulations find that neutering owned cats younger is sufficient to reduce overall population growth rate, regardless of the overall neutering prevalence. Population growth rate is most influenced by owned cat survival and fecundity. Owned cats, which made up the majority of our modelled population, have the most influence on overall population dynamics, followed by stray, feral and then shelter cats. Due to the importance of owned-cat parameters within the current model framework, we find cat population dynamics are most sensitive to shifts in owned cat husbandry. Our results provide a first evaluation of the demography of the domestic cat population in the UK and provide the first structured population model of its kind, thus contributing to a wider understanding of the importance of modelling connectivity between subpopulations. Through example scenarios we highlight the importance of studying domestic cat populations in their entirety to better understand factors influencing their dynamics and to guide management planning. The model provides a theoretical framework for further development, tailoring to specific geographies and experimental investigation of management interventions.

## Introduction

The domestic cat (*Felis catus*) is one of the most popular companion animal species globally. In the UK alone, there are over 10 million owned cats [1–3]. Owned cats form the lion’s share of the UK *F*. *catus* population, but the total population also comprises cats in shelters, and free-roaming, unowned cats that are either feral (largely defined as unsocialised) or stray (largely defined as previously owned and socialised). The overpopulation of unowned cats is often cited as a cause for concern for a range of reasons such as their compromised welfare [4,5], nuisance and public health concerns [5,6] and their potential negative effect on the owned cat population [7] and local wildlife [8,9]. An estimated 250,000 unowned cats live in UK towns and cities [10], approximately 300,000 cats that are not considered pets live on farms [11] and in excess of 150,000 cats enter UK shelters every year [12]. However, these abundances do not currently account for unowned cats living in rural areas outside of farms. Despite clear differences in life history, behavioural repertoires and reliance on humans, these owned and unowned cat subpopulations are interlinked and dynamic, with cats moving and breeding among them. The connectivity between these subpopulations is likely to play a significant role in changes in the numbers of cats residing in each subpopulation as well as the dynamics of the whole cat population.

Domestic cats are highly prolific breeders. The management of domestic cat populations takes a variety of forms around the world, for example through controls on reproduction (such as neutering, indoor-only housing), survival (such as vaccination, care-practices, lethal control) and transitions between subgroups (such as rehoming, straying). Population models can explore the effect on population dynamics of applying interventions or occurrences to any modelled demographic rate, including reproduction, survival and transitions. In the UK, the population management of cats focuses heavily on neutering. In addition to curbing overpopulation [13], neutering of owned cats is encouraged for welfare purposes [14–16] including the prevention of reproductive diseases, reductions in unwanted behaviours and reduced risks of infectious diseases. The timing of neutering is also considered important [17], with many owned cats having accidental litters [18], therefore neutering from four-months is supported by animal welfare and veterinary organisations [19–21]. Additionally, specific goals for population management vary, depending on the local and/or national context, and may include population stabilisation, growth or reduction of any or all the cat subpopulations. The development of a modelling framework is central to understanding the relative impact of differing management and husbandry practices on population-level dynamics, potentially enabling exploration of several competing interventions against desired outcomes at a local and national level.

While many studies have modelled the dynamics of cat populations, very few have integrated across the collective subpopulations of owned, shelter, feral and stray cats. Most have focussed on single subpopulations, predominately either owned [22–24] or feral [25–27] cats and have largely ignored the spectrum of cat subgroups and the movement of cats between them (e.g. stray cats to shelters or owned cats to stray), with the exception of a model specific to North American cities [28]. While the isolated modelling of subpopulations may be appropriate at a localised level where feral cats may be living separate from other subpopulations for example, this is not ecologically appropriate when feral cats live alongside, interact and potentially breed with other cat subpopulations. Isolated subpopulation models do not account for movement of cats in and out of a given subpopulation. Without taking into account the interlinked nature of cat populations, we have little systematic understanding of how subpopulations directly or indirectly influence each other and the impact that interventions on a specific cat subpopulation can have on all aspects of the network.

Here we take an integrated approach to the empirical modelling of the UK cat population, parameterising a Matrix Population Model (MPM) with data-led estimates of vital rates (survival and reproduction) of owned, shelter, feral and stray cats, and estimates of rates of movements and transitions within and among these subpopulations. Through simulations we demonstrate how uncertainty in cat vital rates propagates to form uncertainty in population dynamics. We simulate example localised population management scenarios, particularly increased neutering of owned cats as well as age-dependent neutering of owned cats (commonly suggested methods to help curb overpopulation), to help predict the impact of neutering interventions on population dynamics. We use sensitivity analysis of our models to reveal which vital rates, when altered, have greatest influence on total population growth. We discuss how broad-scale empirical models can help understand owned-unowned population dynamics and predict the impact of management intervention scenarios. With increased data availability, we propose future developments of this modelling approach including consideration of environmental and geographic variation in cat demography and the influence of uncertainty and stochasticity on predictions.

## Methods

Here we explain the structure of our population model, including subpopulations and their links, seasonality and density-dependence. We provide information on the sources of estimates of *F*. *catus* vital rates and describe how uncertainty in these parameters is handled by our modelling. We describe simulation experiments regarding neutering scenarios for population management, then describe our use of sensitivity analyses to reveal the key associations between population growth rates and cat life histories.

### Current knowledge of cat population dynamics in the UK

Most recent estimates suggest the UK is home to around 11 million owned cats [2], with 26% of the human population owning a cat [2]. At a national level, estimates of the owned-cat population have remained relatively stable for the past 10 years [3]. However, populations, especially of stray and feral cats, can vary significantly spatially [10]. The majority of the owned-cat population is neutered [2], with recent estimates collected as part of a UK national survey of Cat Owners [2] suggesting 97% of owned cats to be neutered by the time they are 11 years old. However, this too can vary spatially, with lower rates of neutering in more economically deprived areas and higher rates in affluent areas [29]. Therefore, the overall averaged population dynamics that we see at a national level are underpinned by diverse conditions at localised scales.

### Cat population model

#### Model structure

We developed a multistate Matrix Population Model (MPM), which combines multiple vital rates into an integrative and quantitative measure of population dynamics. These models are widely used to understand and guide management of wild populations, with hundreds of animal demographic studies using this approach [30].

**Fig 1:**
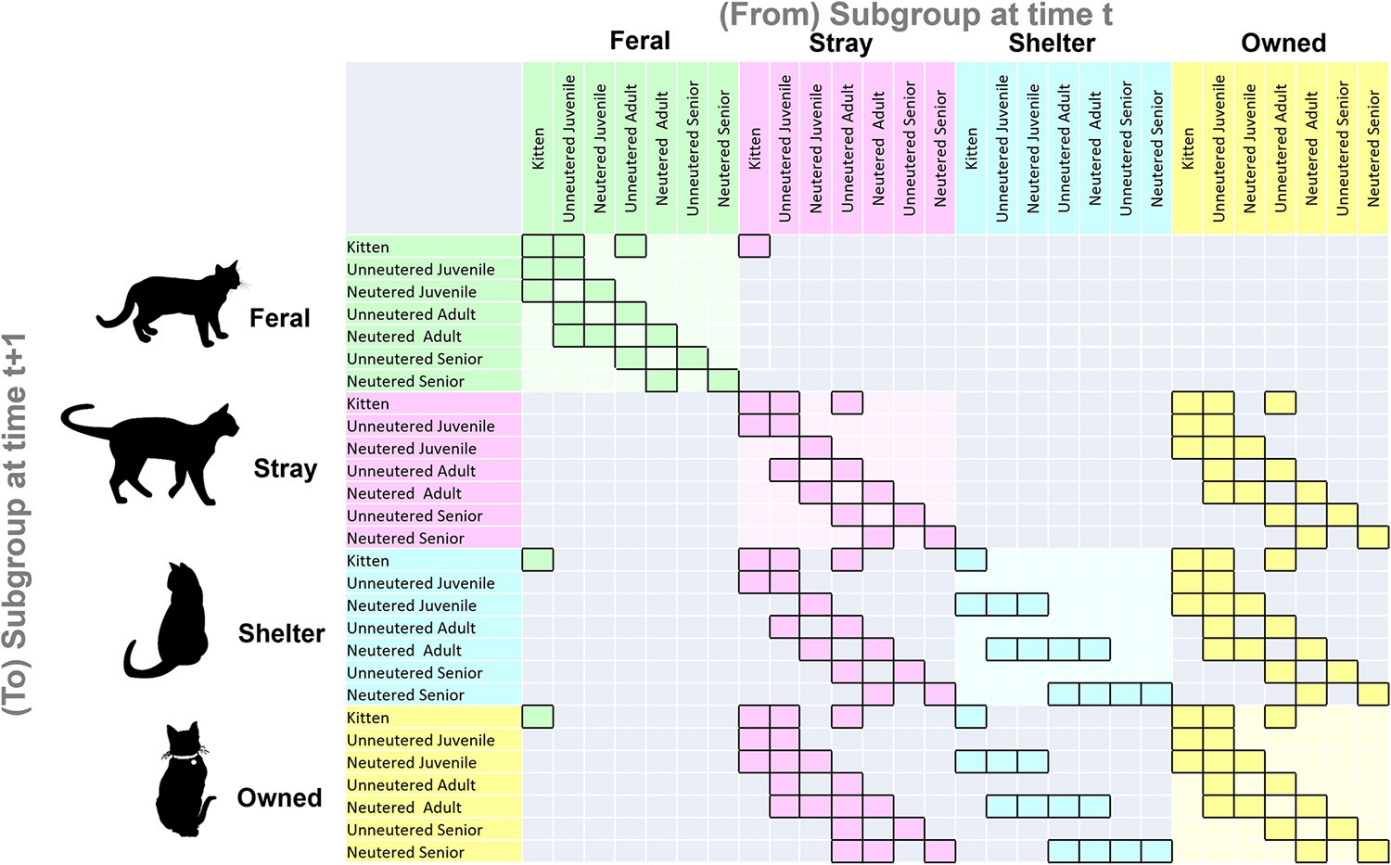
General structure of the model. Outlined cells indicate modelled transitions: Note these transition rates are themselves functions of underlying vital rates (S1–S3 Tables). The larger coloured blocks on the matrix diagonal indicate within-subpopulation population dynamics. The transitions between subpopulations are indicated by the coloured cells outside of the larger coloured squares on the diagonal.

For the purposes of this study, cats are characterised according to four life history stages based on four age stages (kitten, juvenile, adult and senior), four subpopulation types (owned, shelter, stray and feral) and two types of reproductive control (neutered and unneutered), defined below and visualised in Fig 1. For the purposes of this paper, we use the term ‘state’ to refer to the matrix class the cat resides in, i.e. a combination of their age, subpopulation and reproductive status.

Most matrix models only consider females [30], often due to difficulties assigning paternity to males, with the assumption that males do not limit the production of kittens. Therefore, total population size is approximately double if a 50:50 sex ratio is assumed.

#### Age-based stages

Kittens were defined as cats from under 6 months of age [31]. Juveniles were defined as cats from 6 months up to 11 months of age [2]. Adults were defined as cats from 1 year up to, but not including, 11 years. Senior cats were defined as cats 11 years and older [2,31]. These age classes were chosen to enable modelling of age-specific reproduction (see Reproductive Control section) and survival for all subpopulations. We note that due to low survival in feral cats it may be appropriate to exclude the senior age-class, however, it is retained here given the lack of UK-based information on this subpopulation and also to allow for future flexibility in modelling of feral cats. With increased information further age-based stages could be included or removed from the model.

#### Subpopulations

Whilst the dependency of domestic cats on humans for food and shelter, and their degree of socialisation, is most likely to lie on a continuum, discrete categorisation of cats is necessary for modelling purposes, with cats only able to transition between, not within, subpopulations. Our definitions of subpopulations are based on previous academic studies as detailed below.

Feral cats are generally cited as unsocialised to humans, which in the practical sense would typically result in them being fearful of and/or exhibiting defensive behaviour towards people [32–36]. They are thought to be less dependent on humans for intentional provision of resources and more likely to avoid humans [11,37–43]. Consequently, feral cats are much less likely to transition into shelters or into the owned cat population, compared to the stray subpopulation. Indeed, attempting to rehome unsocialised cats or take them into a household would be detrimental to their welfare [44] and is not recommended by cat welfare charities [45–47]. Despite this, it is possible that feral adults are entering shelters either due to difficulties in distinguishing them from stray cats or due to individual shelter practices, for example some shelters may attempt to adopt feral cats if they test positive for FeLV or FIV [48]. However as we do not have any evidence-based figures on rates of feral cats entering into shelters or to domestic homes in the UK, the current model assumes these transitions do not occur. Such transitions could be incorporated in future model iterations where needed subject to sufficient data or when modelling localities where it is known to occur. The exception to no or minimal feral transitions into shelters or into owned subpopulations are feral kittens due to their potentially being considered suitable to be socialised to humans and to live as human companions. While the socialisation window of kittens is considered to end by 7 weeks old, there is a lack of available information on how knowledge around socialisation is applied in practice, hence we accommodate for feral kittens to transition to either shelter or owned subgroups within the kitten stage defined above.

Stray cats are generally considered to be lost, or previously owned, cats [32,38,40,49]. They are therefore typically considered to be socialised to humans and assumed suitable for homing as an owned cat. Consequently, they are generally placed into homes and become owned cats and may be taken into a shelter environment for rehoming. Additionally, if unneutered strays have kittens there is the potential for these offspring to remain unsocialised to humans if they do not experience appropriate human socialisation within the first weeks of their life [50,51]. Stray kittens can therefore also become feral cats. In the current model we assume that previously-socialised stray cats would most likely maintain their classification as ‘stray’, although we cannot completely discount the possibility that cats living as strays for a long time might become avoidant, fearful and untrusting of humans and consequently end up being classed as feral, because they may be assumed to be unsocialised based on their behavioural presentation.

Shelter cats reside in a shelter environment and can originate from feral (as kittens), stray and relinquished owned cat subpopulations. These cats then are homed to become an owned cat.

Owned cats have a close relationship with humans and are closely associated with a household. They are typically considered socialised to humans, dependent on them for resources and regarded as human companions [37,38,40]. Owned cats can come from the stray, feral (as kittens) or shelter subpopulations. They can also feed into the shelter population through relinquishment and can enter the stray population through abandonment or becoming lost.

#### Reproductive control

Cats within each age-stage and subpopulation, with the exception of kittens, are either neutered or unneutered. Although kittens can reach sexual maturity at 4 months, due to the duration of gestation, cats are not expected to give birth until 6 months of age. As breeding in MPMs is defined at the point of individuals being born and not at the point of mating, cats were not assumed to breed whilst kittens. Although we retain the neutered status in senior cats to provide future flexibility in the model framework, as female reproductive success declines with age with peak reproductive activity occurring during adulthood [52], and given this is a female-only model, we currently assume that cats do not breed when senior (i.e. 11 years and older).

For owned cats, prevalence of neutering was first defined at 6-months when cats entered a juvenile stage. This process accounts for a proportion of cats being neutered before six months. As cats can reach sexual maturity [52] and become pregnant at four months [53] with an average gestation period of approximately 65 days [52,54], neutering cats at the six-month transition point within the model accounts for those cats neutered before reaching sexual maturity. Within the juvenile age-stage a monthly rate of neutering was applied. As cats entered the adult age stage a fixed rate of neutering was applied. Due to limited data on the neuter rates of feral cats in the UK, for simplicity we incorporated a fixed rate of neutering upon entering the adult stage only, although there is functionality within the model for neutering to take place at earlier ages. For unneutered stray cats, neutering was modelled as occurring either when within the shelter or upon entering the home environment.

Not all owned cats have outdoor access or mix with other cats. Consequently, for owned cats, we assumed 70% of unneutered cats could get pregnant, based on estimates of outdoor access [55,56], although this does not account for the unknown numbers of unneutered indoor-only cats, which can also reproduce when mixed-sex groups are housed together. Per the literature, we included reduced fecundity for feral and stray cats [57], with fewer viable kittens likely due to a poorer body condition or body weight. For all subpopulations we assumed reduced birth rate when juvenile relative to adult, due to reduced litter sizes and accounting for average reproductive maturity after six-months. Already pregnant shelter cats were assumed to give birth after arrival at the shelter during the first month, therefore had the fecundity of their origin subpopulation. However, the model assumes a reduced birth rate with earlier stage pregnancies terminated via pregnant spays [53], which is often considered the most appropriate option given that giving birth and raising kittens in care has negative implications for the welfare of the queen and her kittens [58]. See S1 Table for all parameters and their derivation.

#### Data

The underlying model structure required estimates of 40 parameters including survival rates (φ) for each state, these are based on the proportion of cats that do not die (euthanasia or otherwise) in each time-step, fertility rates (ψ) for each state capable of reproduction and transition probabilities (ω) for each state that can move into different subpopulations (S1 Table).

Prior to model development, an extensive literature search of primary and secondary literature was carried out to pool together known information from over 40 academic and animal welfare sources. Survival parameters were converted into an approximate monthly rate using the formula p~1-(1-p)^1/n^, where n = the number of months the original parameter was recorded over. Where data were unavailable, informed estimates based on the expected behaviour of the system were included.

The full list of parameters and estimates are in S1 Table. Parameters underlying the matrix entries and associated formulae are available in S1 and S2 Tables.

#### Initial stage structure

Initialising the model requires the number of cats in each state. A consistent stage-structure enables us to focus on the impact of demographic interventions, rather than the influence of the stage-structure itself. We approximate an initial stage distribution, based on our current understanding of feral and stray cat populations, from previous research in this area [10,11]. We assumed that 69% of free-roaming unowned cats were feral based on current unpublished data on outcomes for unowned cats within urban areas undergoing community neutering campaigns (e.g. [59]), with studies in other areas similarly estimating between 66% and 75% of unowned cats to be feral, rather than socialised strays [35,57].

The number of cat shelters in the UK was approximated from CatChat [60], an independent web-based charity that lists cat homing services in the UK and Ireland [accessed 23/09/2021], where we found 1036 cat rehoming services in the UK and cat numbers were then extrapolated based on current understanding of the average number of cats per shelter [61]. It is notable that although significant numbers of cats are thought to enter shelters yearly [61,62], their limited capacity and the typical short term or temporary nature of shelter housing means the numbers residing at any given time is likely to make up the smallest cat subpopulation with a high throughput due to population turnover.

Owned-cat estimates were based on data generated from a recent nationally representative survey of cat owners [2], and were similar to other studies [1] and industry estimates [3]. Given this is a female-only model, we assumed the sex ratio to be equivalent across these populations, such that the relative numbers in each subpopulation reflects the relative numbers of females within each subpopulation.

Generally, we approximate within our starting stages that 92.2% of the UK cat population are owned, 0.2% are in a shelter, 2.3% are stray and 5.2% are feral. However, we note that at a local level these proportions are likely to vary considerably. See S1 Table for starting subpopulation sizes.

#### Seasonality

Female cats exhibit seasonal breeding patterns with pregnancy rates highest in Spring and lowest in Winter [63]. The MPM allowed us to take a probabilistic approach to seasonal reproduction in cats, with reproductive parameters governed by a monthly probability of parturition. We assume 78% of litters are born in Spring and Summer and 22% in Autumn and Winter, equating to 13% and 3.7% probability of monthly litters respectively, approximately similar to published pregnancy studies [63].

#### Density-dependence

The sizes of the owned and shelter populations are limited by the number of available spaces in homes and shelters respectively. We implemented a density dependent feedback mechanism in the cat population by specifying a carrying capacity for both the owned and shelter subpopulations which when approached alters the transitions into and out of the subpopulations. The carrying capacity was representative of the housing constraints imposed on owned and sheltered cats. Parameter values were set during the model development stage to promote a relatively stable environment during average model runs to enable intervention testing. While the carrying capacities are somewhat arbitrary and curb growth rather than prevent exceeding of limits, the functions act to prevent unrealistic geometric growth of homed and sheltered subpopulations, meanwhile accounting for the interlinked nature of the cat subpopulations.

We set the carrying capacity of owned cats to approximately 10% above the starting population, given national owned-cat estimates have not exceeded this in the past 10 years [3]. Above this point, we assume a change in the transition rates, with owned cats twice as likely to move to a stray subpopulation, due to likely abandonment or increased likelihood of cats roaming and becoming lost that may occur at high densities. Although research is limited in this area, too many cats in the household is often cited as a reason for abandoning a cat [64] and anecdotally thought to increase straying [65]. When the owned-cat estimates reach carrying capacity, we also assume a significantly reduced capacity to take in feral and stray cats, with a 100-fold reduction in the proportion of feral and stray cats that transition into the owned subpopulation. Additionally, as owned-cat populations start to approach this carrying capacity (from 1% above the starting population) we assume a 25% increase in owned cats moving to a stray subpopulation and a 75% and 55% decrease in the probability of a feral or stray cat being taken in, respectively. Although not included, we cannot discount that owned cat mortality increases at greater owned cat densities. Given the limited data, we increase the proportion of owned cats that are abandoned or lost, which in terms of modelling the total population is functionally equivalent to increasing mortality, given that upon entering the stray subpopulation their mortality increases. However, if the model’s density-dependent mechanism is incorrect the outcome will be that stray populations may be overstated within the current framework. Consequently, this density-dependent process should be re-evaluated in the light of emerging evidence or if the model is to be applied to other areas.

We set shelter capacity to approximately 10% above starting estimates, given that median numbers housed may be less than maximum capacity, for example an earlier study [12] estimated the median number of housed cats per shelter to be 27 with a maximum capacity of 30. Above this point we assume a 75% reduction of the normal feral and stray cats’ intake and owned-cat intake was reduced to 50% of normal intake. Additionally, as shelter cat populations approach carrying capacity (1% above the starting estimate) we assume intake of feral, stray and owned-cat intake reduced by 25%. We currently do not include decreased survival (which would allow for increased euthanasia) when shelters are at capacity. Studies to date have found most UK cat rescues hold a waiting list to manage the demand on intake [12] with over-capacity not cited as a reason for euthanasia in cat populations [66]. However, further research is needed into decision-making regarding euthanasia within UK shelters and such density-dependent feedback could be incorporated within this model framework if appropriate.

These density-dependent feedback mechanisms are calculated at every time-step of our model projections.

The current model imposes no limits in terms of carrying capacity on feral and stray subpopulations. With lower numbers in these subpopulations, at a national level they may not be residing at capacity; however in areas where they live at higher densities they will be inhibited by environmental constraints, although the precise nature of these constraints are unknown. Additionally, when these populations increase it is unclear whether or how the proportion of these cats that get rehomed or neutered are affected. Therefore, any assumptions would be too speculative and beyond the scope of this study but could be included within the model framework when further information becomes available.

### Model projections and uncertainty

The model projects a given population structure forward in time to a new projected population size and structure. The basic formulation of the demographic model is:

$$n_{t+1}=An_{t}$$

Where **n**_**t**_ is the vector of the number of cats in each state at time t and **A** is the projection MPM composed of survival, fecundity and transitions for each state.

To allow flexibility in the modelling approach, such as incorporating seasonality, and to simply incorporate the process of cats entering and leaving shelters, a monthly time-step was modelled. The median length of stay in a shelter was approximated to be 45 days similar to published estimates [67,68], hence modelling with timesteps longer than one month would prevent accurate modelling of the shelter subpopulation, either artificially increasing the average time cats spent in shelters beyond that observed in the UK or preventing the inclusion of a shelter subpopulation altogether.

To account for the uncertainty in survival parameters when projecting population dynamics we apply a simulation analysis, which allows testing of different parameters for each simulation. All values from relevant literature were considered, and simulated values were chosen for the mean and variation of survival rates to span the published averages (S1 Table). Survival parameter values were randomly simulated from a beta distribution with relevant measures of mean and variance.

While individual cats and litters vary dramatically in litter size, published population estimates generally agree on a typical mean litter size (see S1 Table). For modelling purposes, we applied a measure of average litter size for each cat type, with natural seasonal variation applied as detailed previously.

We also assumed transitions between states remained constant, with owned cat neutering rates forming part of our simulation experiments and transitions of cats in and out of homes and shelters varying due to constraints imposed by the density-dependent processes detailed above. See S1 Table for full list of parameters.

### Model validation and application

Given the stability of the owned-cat population over the past 10 years [3], we may expect national dynamics to be relatively stable, with population growth approximating one, even if localised dynamics are variable. Therefore, model checks included relative stability in dynamics and projected stage-structure not too dissimilar from initial starting conditions under average conditions explored during deterministic model runs. These checks assure a relatively stable baseline model for the testing of different scenarios and management interventions.

### Simulation experiments

We simulated 33 different possible localised scenarios across two broad simulation experiments, with a focus on neutering interventions. As the model is female-specific, our neutering scenarios refer to neutering females only.

Our first simulation experiment explored the effect of overall owned-cat neutering rates across three different scenarios. Local and regional neutering rates are likely to vary considerably, for example due to local interventions or due to other human-social factors relevant to pet keeping. Owned-cat neutering rates are thought to be lower in socio-economically deprived areas relative to more affluent areas [29], which in turn is hypothesised to influence the number of stray and feral cats. We test whether overall owned-cat neutering rates alone could drive differences in the population growth and stage structure of unowned-cat subpopulations. We illustrate differences in population dynamics across three scenarios: low (90%), medium (95%) and high (98%) adult owned-cat neutering prevalence, which approximate low (83%), medium (88%) and high (92%) overall owned-cat neutering prevalence.

Our second simulation experiment tested the efficacy, in terms of reduced population growth, of neutering owned cats prepubertally compared to later-age neutering. We constructed models that simulated different degrees of neutering prior to 6 months, whilst maintaining a constant adult neutering prevalence. In doing so, we test whether the timing of owned-cat neutering, not neutering itself, affects population growth rate. We increased the proportion of kittens neutered for each scenario across 10 values between 0.05 and 0.5. We ran this for populations that had both high (98%), medium (95%) and low (90%) adult neutering prevalence, equating to 30 different scenarios.

Each localised scenario was initialised with an arbitrary starting population of 100,000 cats, which was chosen to enable modelling of meaningful numbers of cats in the smallest subpopulation of cats in shelters. We modelled the population trajectory over 10 years under different scenarios to identify potential population growth rate and stage structures. We applied a deterministic approach using average matrices for each scenario, although still subject to seasonality and density-dependent processes. We then ran each scenario via our uncertainty simulations, to calculate median and 95% confidence bounds for population growth and population size, with the 95% confidence intervals (CI) calculated as the 2.5% and 97.5% quantiles of 20,000 simulations.

### Sensitivity analysis

To understand the impact of vital rates on population growth we applied a sensitivity analysis. Whilst matrix sensitivity analysis is usually based on determining the sensitivity of the asymptotic growth rate to changes in matrix elements, such analysis assumes that the population grows at a constant rate, whereas our modelling approach contains nonlinear functions including seasonality and carrying-capacities. Consequently, in order to gain proper knowledge on the influence of underlying vital rates on population changes we numerically calculated the influence of changes to underlying vital rates to projected population growth rate.

Specifically, we reduced each vital rate by 10%. We used a transformation approach to account for dissimilar demographic scales of fecundity rates (which can have any positive value) and survival and transitions that are probabilities so must lie between zero and one. Consequently, to generate a 10% reduction in vital rates, values were calculated on the log-transformed fecundity rates and logit-transformed survival and transition rates. These were then back-transformed by the exponential function and inverse-logit function respectively. This approach has been found to successfully stabilise the structural relationship between the mean and variance both within and across demographic categories [69,70], meaning any adjustments are comparable.

Following each adjustment to a vital rate, we simulated the dynamics of cat population from the same starting stage structure (as detailed previously), running the model for 120 time-intervals, to calculate the 10-year projection, and consequently an altered population estimate (N_altered_). The proportional change in population growth rate (PGR) due to the reduction in vital rates is then estimated as

$$(N_{unaltered}‐N_{altered})/N_{altered}$$

All models were specified within R version 4.0.2 [71].

## Results

### Average model: Proportion of owned cats neutered

**Fig 2:**
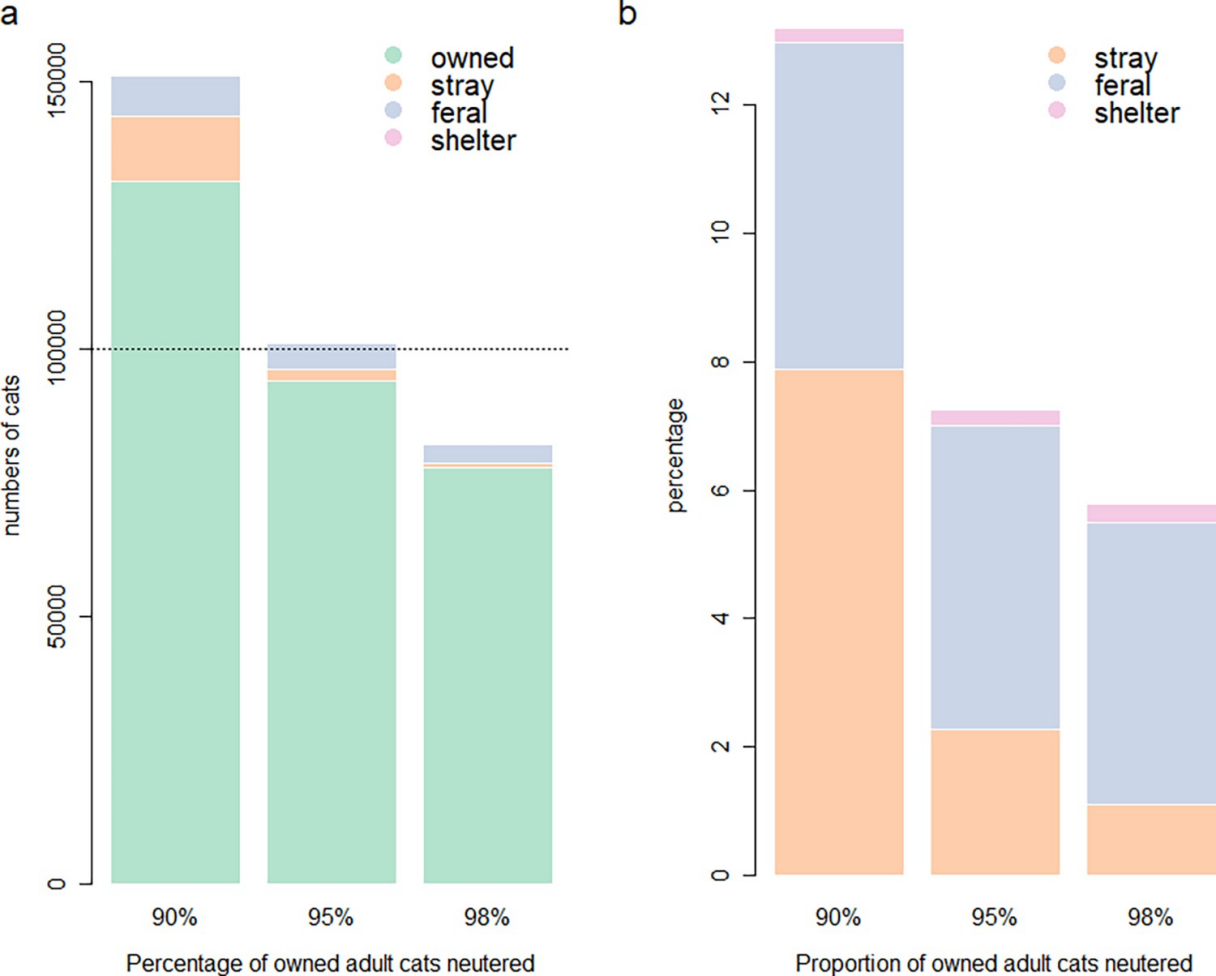
a) Absolute numbers of cats in each subpopulation under different owned-cat neutering practices. Shelter cats are not as visible due to the low proportion that reside in this subpopulation. b) percentages of cats in the unowned—stray, feral and shelter subpopulations under different owned-cat neutering practices following a 10-year projection assuming a starting abundance of 100,000 cats (indicated by the dashed line).

The projected population growth rates obtained from the three scenarios with low (90%), medium (95%) and high (98%) projected adult owned-cat neutering prevalence are shown in Table 1 and Fig 2. Under average conditions the long-term growth rates for all subpopulations were closest to one with total PGR approximately stable over a ten-year period (PGR = 1.01; Table 1), thus providing a relatively stable environment under average model conditions to enable intervention testing. When the prevalence of neutering among owned adult cats decreased by 5% from the average, mean population growth rate values for all subpopulations were greater than 1, with an average overall population increase of 151%, with the most notable increase in the stray subpopulation (Table 1). When the proportion of adult owned cats neutered increased by 3% above average, projections for all subpopulations produced mean population growth rate values less than 1, with an average overall population decline of 18% (Table 1).

**Table 1:** Projected population growth rate (PGR) of cat subpopulations under different neutering strategies for owned adult cats.

Scenario	Total PGR	Owned PGR	Stray PGR	Feral PGR	Shelter PGR
90% adult owned cats neutered	1.51	1.42	5.07	1.63	1.21
95% adult owned cats neutered	1.01	1.01	1.00	1.01	1.00
98% adult owned cats neutered	0.82	0.84	0.39	0.77	0.83

Additionally, at lower rates of owned-cat neutering, whereby overall neutering of adult owned cats was reduced by 5%, the proportion of the population in the stray subpopulation increased to 7.8% of the total population, whereas under medium owned-cat neutering conditions they were only 2.3% (Fig 2).

### Average model: Influence of age of owned-cat neutering

**Fig 3:**
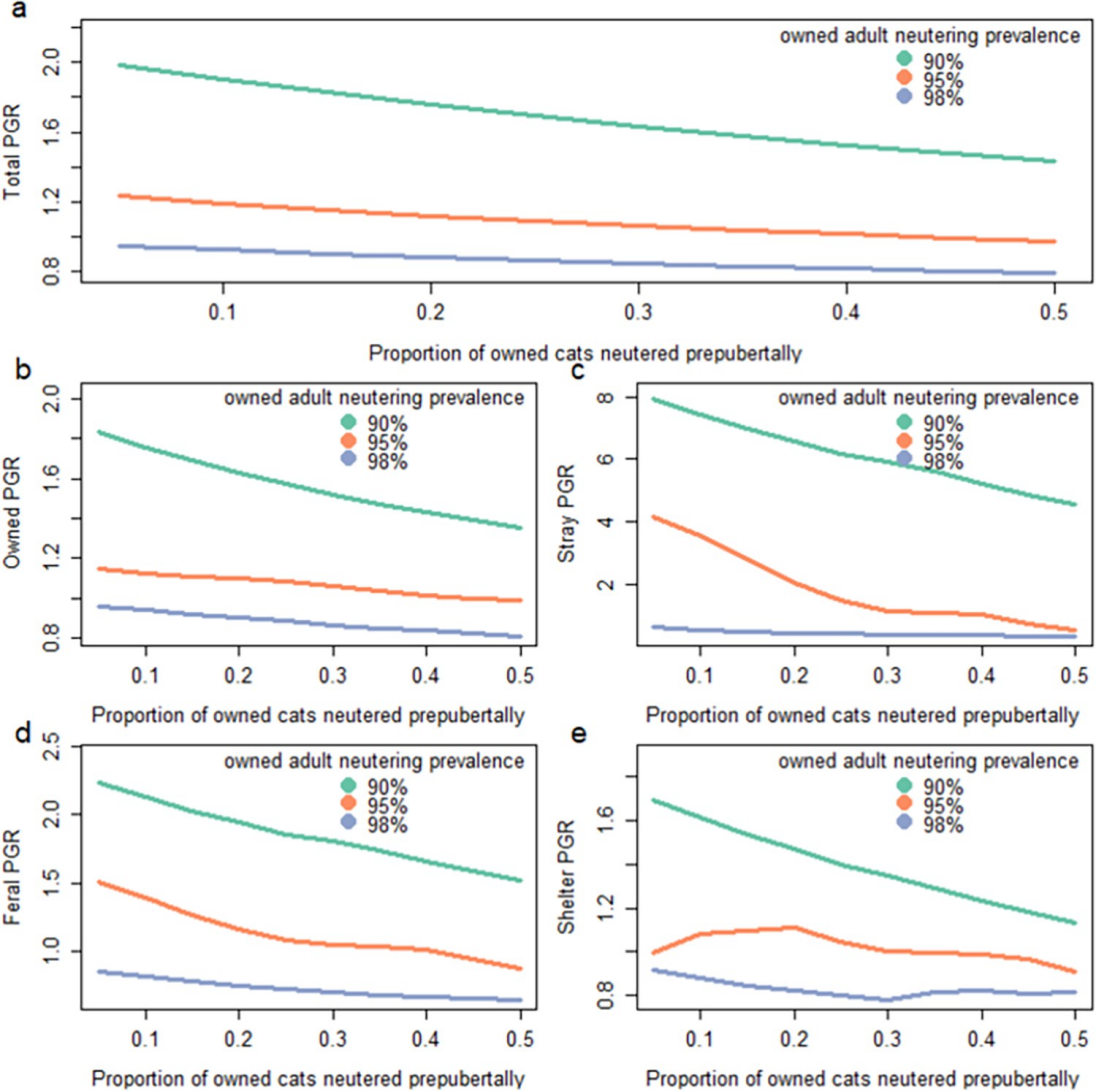
The ten-year population growth rate (PGR) for (a) the total cat population along with (b-e) subpopulations relative to the proportion of cats that are neutered prepubertally, holding overall adult neutering prevalence constant at either low (90%), medium (95%) or high (98%) prevalence. All scenarios had the same starting stage structure and starting abundance of 100,000. Note the different scales for population growth rates which were used for visualisation purposes.

Increased rates of prepubertal neutering of owned cats, ranging from 5 to 50% of all cats neutered before six months, resulted in reduced population growth under a range of neutering scenarios. Increased levels of prepubertal neutering resulted in reduced population growth, despite overall adult owned-cat neutering prevalence remaining constant. The influence of prepubertal neutering in populations of cats with low overall neutering prevalence was particularly profound (Fig 3).

**Fig 4:**
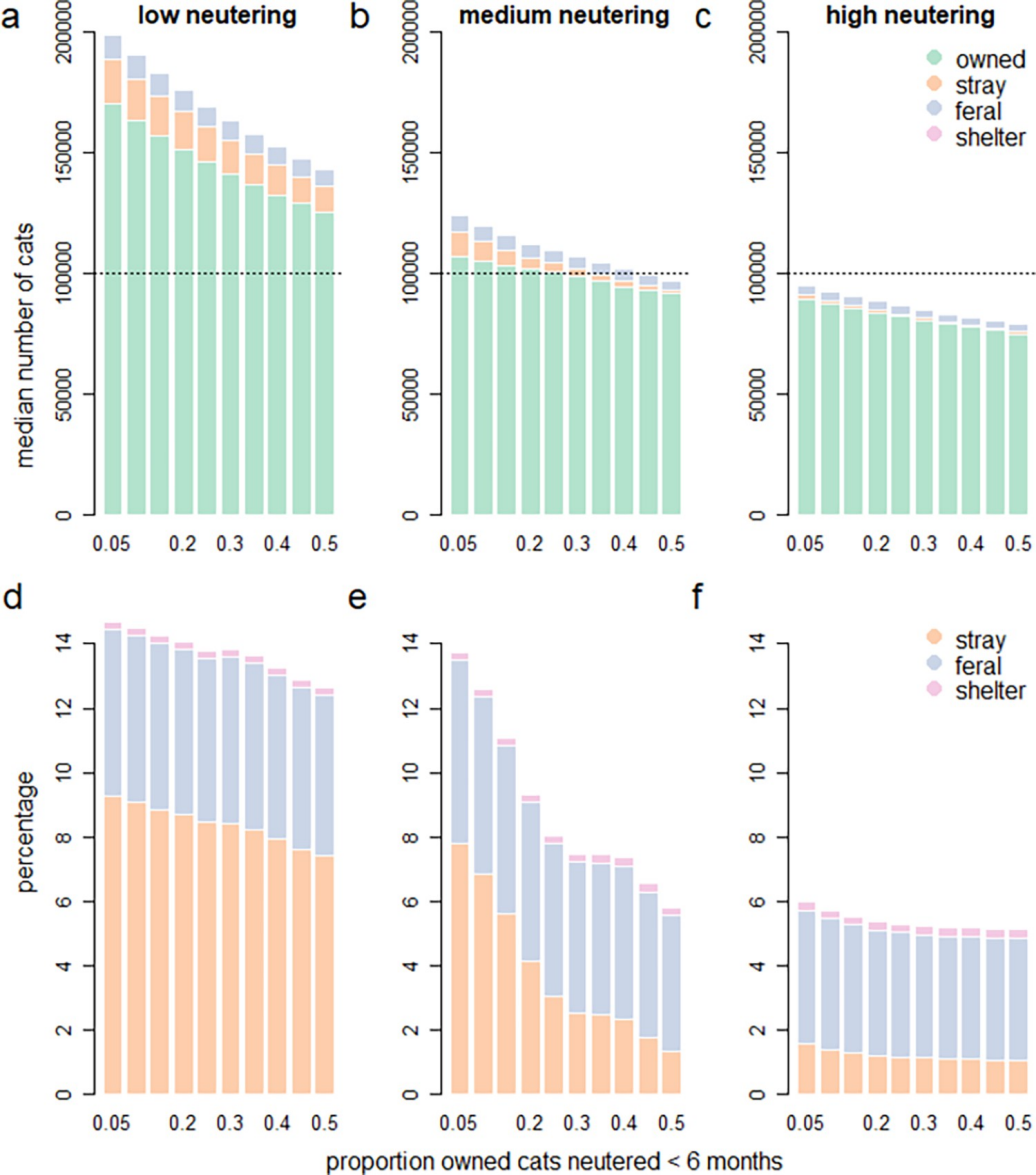
(a-c) The absolute numbers of cats that reside in each subpopulation. Shelter cats are not as visible due to the low proportion that reside in this subpopulation. (d-f) The percentages of cats in the unowned- stray, feral and shelter subpopulations following ten-year projections of populations with low (90%), medium (95%) and high (98%) proportions of the adult cat population neutered, under scenarios where between 5% and 50% of the owned cats are neutered prepubertally to prevent parturition at 6 months. Each simulation assumed a starting abundance of 100,000 cats (indicated by the dashed line).

The proportion of stray cats in the population reduced with increased proportions of owned cats neutered prior to six months. This effect was greatest under average neutering prevalence whereby 7.8% of the population were projected to be stray when just 5% of owned cats were neutered prior to six months, dropping to 1.3% when 50% of the owned-cat population were neutered prior to six months, despite the proportion of owned adult cats neutered remaining constant (Fig 4).

### Uncertainty simulations

#### Simulation experiment one: Proportion of owned cats that are neutered

**Fig 5:**
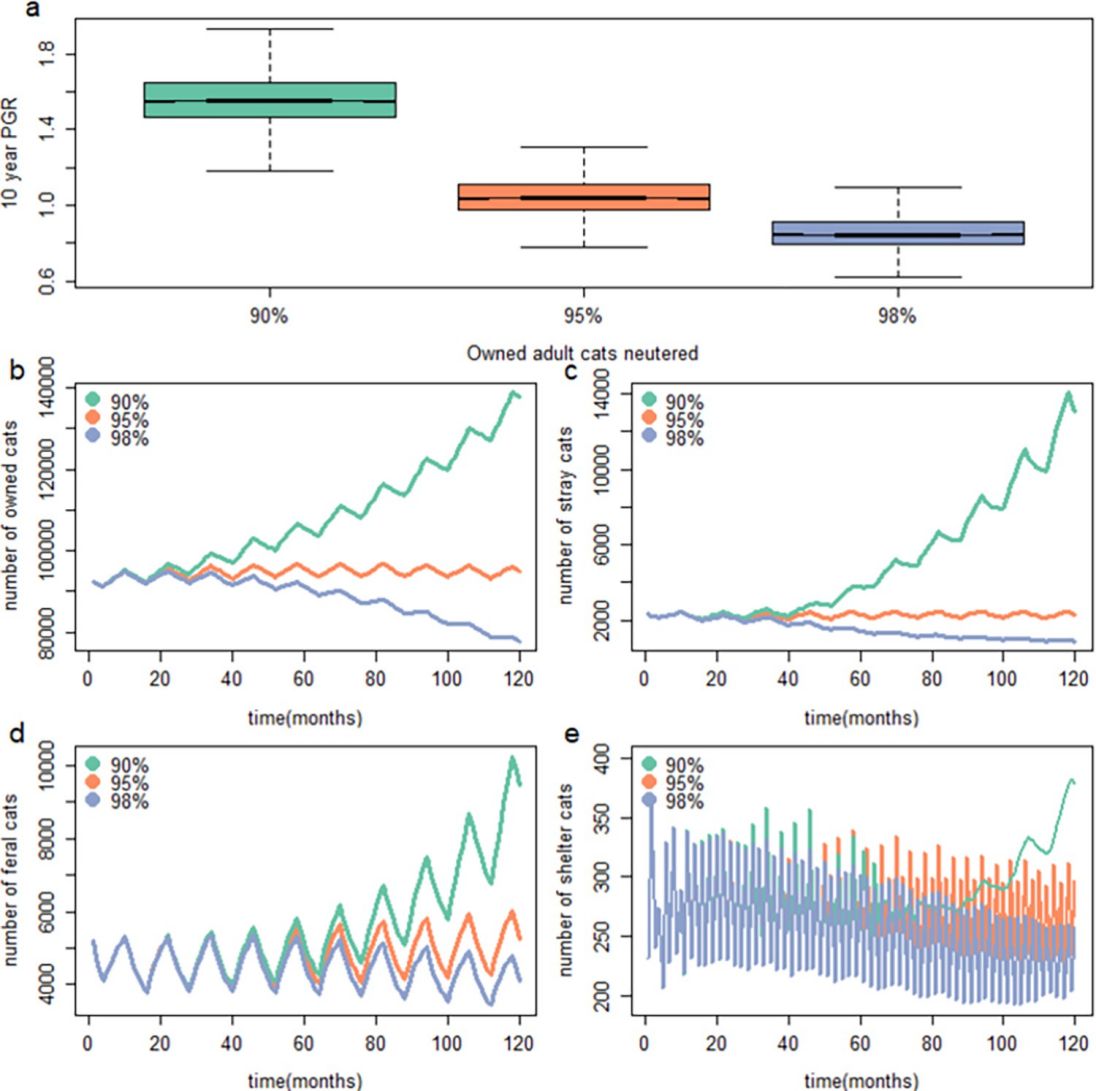
Effects of adult owned-cat neutering prevalence on (a) the 10-year population growth rate (PGR) and (b-e) the median population dynamics for each cat subpopulation assuming the same starting stage structure and starting abundance of 100,000 cats. Note the different scales for population size which were used for visualisation purposes.

Uncertainty in estimates and the mean projected population growth rates obtained from the three modelled scenarios with low (90%), medium (95%) and high (98%) adult owned-cat neutering prevalence are shown in Table 2 and Fig 5. At low neutering prevalence among owned adult cats, median population growth rate values for all subpopulations were greater than 1, with an average overall population increase of 155% (Table 2), with the most notable increase in the stray subpopulation (Table 2: Fig 5C). Projected population growth rates were lowest for all subpopulations under scenarios with high adult owned-cat neutering prevalence, with an average overall population decline of 5% (Table 2).

**Table 2:** Median 10-year population growth rate (PGR) projections (95% CI) according to levels of neutering of owned cats.

Scenario	Total PGR	Owned PGR	Stray PGR	Feral PGR	Shelter PGR
90% adult owned cats neutered	1.55 (1.31, 2.04)	1.42 (1.27, 1.57)	5.17 (2.91, 8.80)	1.77 (0.52,10.19)	1.26 (1.01, 1.67)
95% adult owned cats neutered	1.04 (0.88, 1.49)	1.02 (0.91, 1.09)	1.01 (0.43, 2.86)	1.07 (0.16, 8.66)	1.01 (0.82, 1.10)
98% adult owned cats neutered	0.85 (0.71, 1.29)	0.85 (0.73, 0.97)	0.40 (0.31, 0.64)	0.86 (0.10, 7.51)	0.83 (0.80, 0.99)

Additionally, at low prevalence of neutering owned adult cats, the predicted abundance of stray cats increased over five-fold relative to the medium-neutering model (5.7; Figs 5 and S1). In this scenario the proportion of the population in the stray subpopulation increased to 8.2% of the total population over a ten-year simulation, whereas under medium owned-cat adult neutering conditions they were on average 2.2% (S1 Fig). The results show how differences in owned-cat management can have profound impacts on population dynamics of all cat subpopulations.

#### Simulation experiment two: Influence of age of owned-cat neutering

**Fig 6:**
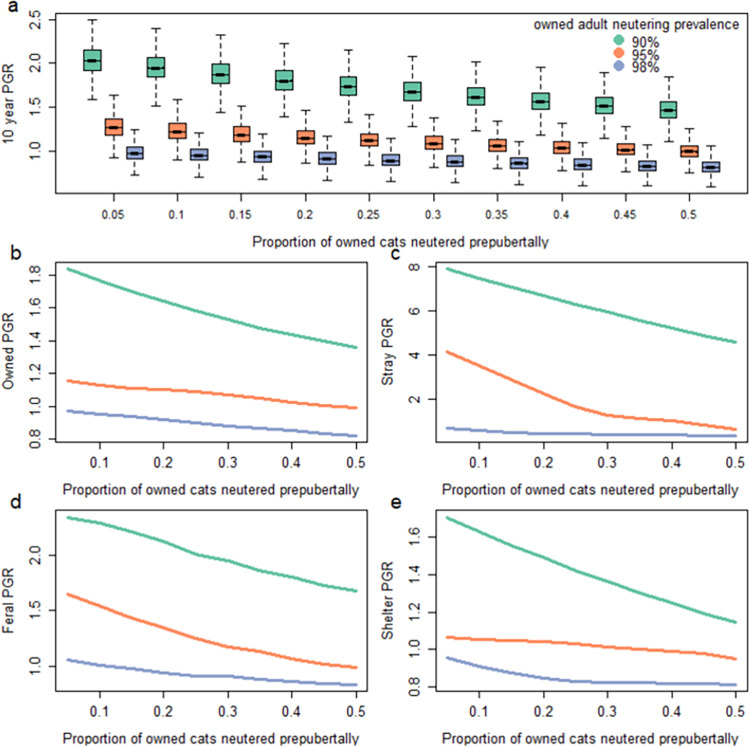
Median ten-year population growth rate (PGR) for the (a) total cat population and (b-e) cat subpopulations relative to the proportion of cats that are neutered prepubertally, holding overall adult neutering prevalence constant at either low (90%), medium (95%) or high (98%) prevalence. All scenarios had the same starting stage structure and starting abundance of 100,000 cats. Note the different scales for population growth rates which were used for visualisation purposes.

Timing of neutering can have a significant impact on the population dynamics of cat populations and their subpopulations (Fig 6, Table 3). Increased neutering of cats prepubertally to prevent parturition at 6 months resulted in a decrease in median total population growth rate at high, medium and low neutering proportions of cats neutered prepubertally, despite adult neutering prevalence remaining constant (Fig 6A, Table 3).

**Table 3:** Median 10-year population growth rate (PGR) projections (95% CI) according to proportion of owned cats neutered prior to 6 months to prevent parturition at 6 months (prepubertally) and overall levels of neutering of adult owned cats.

Scenario	Proportion cats neutered prepubertally	Total PGR	Owned PGR	Stray PGR	Feral PGR	Shelter PGR
Low overall adult owned-cat neutering prevalence (90% neutered)	Low (5%)	2.02 (1.73, 2.54)	1.84 (1.63, 2.03)	7.91 (5.06, 12.56)	2.34 (0.85, 11.09)	1.74 (1.36, 2.26)
	High (50%)	1.47 (1.24,1.97)	1.36 (1.22, 1.49)	4.61 (2.50, 7.94)	1.68 (0.45, 10.07)	1.17 (1.00, 1.56)
Medium overall adult owned-cat neutering prevalence (95%)	Low (5%)	1.27 (1.07, 1.77)	1.16 (1.07, 1.26)	4.16 (1.19, 8.07)	1.65 (0.39, 10.18)	1.08 (0.99, 1.46)
	High (50%)	0.99 (0.84, 1.43)	0.99 (0.87, 1.08)	0.65 (0.40, 1.78)	0.98 (0.13, 8.13)	0.97 (0.81, 1.08)
High overall adult owned-cat neutering prevalence (98%)	Low (5%)	0.98 (0.82, 1.45)	0.97 (0.85, 1.05)	0.71 (0.40, 1.95)	1.06 (0.16, 9.04)	0.97 (0.81, 1.08)
	High (50%)	0.82 (0.69, 1.25)	0.82 (0.71, 0.94)	0.38 (0.30, 0.54)	0.83 (0.09, 7.25)	0.83 (0.80, 0.96)

Similarly, declines in the average population growth rate were observed for all subpopulations with increased prepubertal neutering (Fig 6B–6D).

Prepubertal neutering resulted in reduced absolute population size for all the subpopulations relative to scenarios where fewer cats were neutered prepubertally (S2 Fig). Additionally, the relative proportion of the population that are stray was greatest when there was just 5% uptake of prepubertal neutering of owned cats (9%, 7% and 2% for the low, medium, high neutering scenarios respectively) and is lowest when there is 50% uptake of prepubertal neutering of owned cats (7%, 2% and 1% for the low, medium, high neutering scenarios respectively; S2 Fig).

### Sensitivity analysis

**Fig 7:**
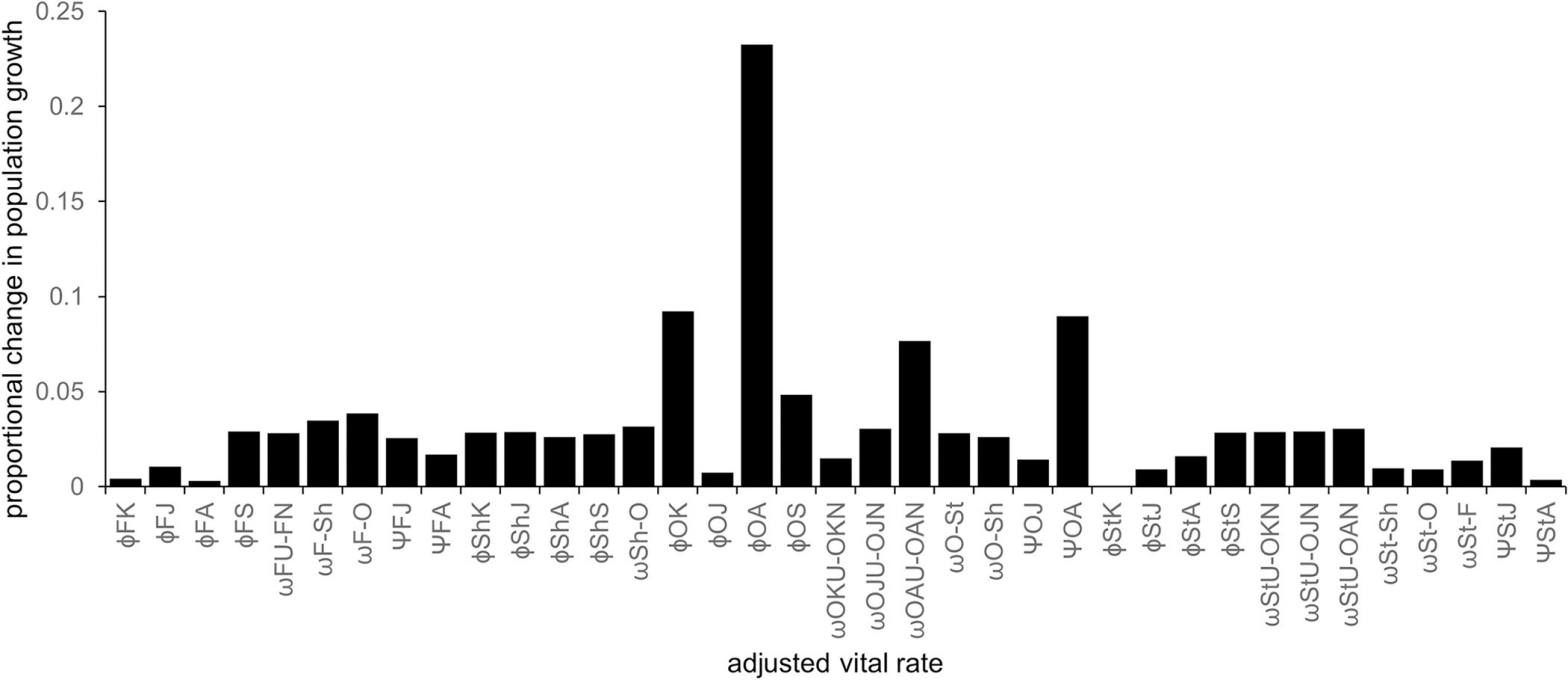
Sensitivity of the projected population growth rate to survival (ɸ), fecundity (Ψ) and transition (ω) parameters, for feral (F), shelter (Sh), owned (O) and stray (St) cats of different age classes kitten (K), juvenile (J), adult (A), senior (S) and neuter rates (U-N).

The sensitivity of each vital rate to overall population growth rate is assayed by the proportional change in population growth rate accounted for by a 10% reduction in the underlying vital rate. The survival of owned adult cats was most influential on overall population changes, followed by the survival of owned kittens and birth rate of owned adult cats (Fig 7). We found that the vital rates of owned cats, including transitions out of the owned-cat subpopulation into other subpopulations were most influential on population growth, totalling a 66% change to population growth rate, followed by the demographics of stray (20%) and feral (19%) cats. The demographics of shelter cats were least influential on population growth (14%).

## Discussion

The interconnected nature of cat populations is often neglected in domestic cat population models, but our results provide strong evidence that their links can significantly influence their predicted dynamics and abundance. We found reproductive control of female owned cats influences all cat subpopulations, with reduced neutering of owned cats resulting in population booms, especially in the stray cat subgroup. This highlights one way in which owned cat husbandry, and consequently human behaviour, is a key contributor to unowned cat populations, with straying or abandonment of unneutered cats and unwanted litters providing a persistent source of unowned cats in the environment. Across the UK, there is expected variation in the proportion of owned cats that are neutered, in part due to socio demographic factors [29]. Initial insights from our example neutering scenarios indicates that localised neutering conditions can result in shifts in population dynamics, with average growth rates dependent on the proportion of female owned cats that are neutered and their age of neutering. Our study also illustrated that the growth of the population was highly sensitive to the owned-cat subpopulation. Together our results demonstrate the importance of considering demographic connectivity within cat populations where subpopulations co-exist. Further, we have shown the adaptability of the framework to reflect localised management scenarios i.e. different neutering practices.

Our model accounted for age, reproductive stage and subpopulation for all cats, resulting in a 28-state model. We accounted for deficiencies in data availability by modelling uncertainty in survival estimates, computing outcomes for a large number of simulated matrices in which the survival rates were sampled randomly between predetermined limits. Despite these constraints on data availability, our modelling approach generated an average ten-year population growth rate that approximated one, commensurate with the stability observed in the abundance of owned cats, nationally [3], providing a stable baseline model for the testing of different scenarios. Additionally, the effect of uncertainties associated with the parameters were addressed using a global sensitivity analysis, which provided a means to retrospectively determine which of these parameters, if incorrectly specified, has the greatest impact on model outcomes. This approach provides a framework to explore the relative influence of interventions and to examine intrinsic characteristics of the entire population.

Our scenarios showed that overall owned female neutering rates, and the proportion of these cats neutered prepubertally, can influence the proportions and abundances of cats residing in each subpopulation. There is evidence that the abundance of unowned cats (feral and stray) is variable across the UK’s urban landscape [10]. Our scenarios highlight one mechanism by which this may occur: as fewer female owned cats are neutered, the absolute numbers and the proportion of the population residing in the stray and feral subpopulations increase. Indeed, earlier studies have found that in affluent areas, where the proportion of owned-cats neutered are anticipated to be high [29], there are fewer unowned cats [10,72,73]. Given the same starting population structure, changes to the owned-cat neutering parameters alone can result in very different population structures and abundance of domestic cats in localised environments.

The timing of neutering is increasingly considered an important factor to prevent accidental litters and consequently overpopulation [53,74]. Although unusual, female kittens can become pregnant at four months [52,53]. Despite this, a high proportion of owned cats are not being neutered until six-months or later after the cats have typically reached sexual maturity [2,75] increasing the risk of unplanned litters, with an estimated 80% of owned-cat litters accidental [18]. Consequently, prepubertal neutering of owned cats from 16 weeks is commonly supported by cat welfare and veterinary organisations [19–21]. We found the age that owned cats were neutered could have profound impacts on all cat subpopulations, despite adult neutering prevalence remaining constant. Population growth rates and absolute abundance decreased when a higher percent of cats were neutered to prevent parturition occurring at six months. Additionally, in simulations where the proportion of adults neutered were generally higher, the proportion of owned cats neutered prepubertally influenced the relative proportion of free-roaming stray and feral cats. This indicates even in areas with high adult neutering prevalence, populations can still grow in scenarios where most owned cats are neutered after a possible age of breeding, resulting in increased litters and ultimately increased proportions of unowned cats. These findings question the validity of benchmarking the management of cat populations using overall prevalence of owned-cat neutering alone, with timing of neutering an underpinning factor that could influence whether a population is in growth or decline.

Our modelling approach, and consequently neutering scenarios, was female-only. Routine neutering of both male and female cats is important to avoid unwanted reproductive activity, associated undesirable behavioural problems and disease transmission. However, there is no evidence that males are the limiting factor for domestic cat populations given a single unneutered male can potentially fertilize multiple unneutered females, suggesting focusing on the female segment of the population within the modelling approach is justified, particularly when considering reproductive interventions. However, a female-only model prevents assessment of other factors, for example the impact of sex-specific demographic rates, such as males likely having lower survival rates [15,76] or the wider implications of neutering male cats, which may influence survival rates, such as through reductions in conflict, competition, and infectious disease [77].

Estimates of population growth were more sensitive to the demographics of the owned-cat population. Therefore, increased neutering of owned cats may have the greatest influence on population growth, especially if paired with reductions in the abandonment and losses of cats into the stray subgroup. Accidental unplanned litters in owned cats may be particularly problematic, resulting in increased numbers of unneutered cats in areas where neutering rates are low. This highlights the importance of neutering and its links to the stray subpopulation and explains why we observed significant changes in the stray population under different owned-cat neutering practices.

### Future directions

Whilst the modelling framework recognises uncertainty in demographic traits, further work is required to minimise uncertainty of estimates. Current data limitations, including data gaps and data quality, prevented us from untangling environmental variability (i.e. naturally occurring variation through space and time) from demographic uncertainty (i.e. errors due to field sampling and/or extrapolation to the UK situation). With further UK-based data we could incorporate environmental variability and reduce parameter uncertainty. An advantage of this model is that it can be easily modified to update model parameters as new information becomes available and for implementation in different geographical areas where cat husbandry may vary, such as neutering rates highlighted here, but also any husbandry practice that affects vital rates. Parameters within the framework can be adapted to include locally measured vital rates, rates of transition, carrying capacities (which may also vary through time) and forms of density-dependence. Additionally, in this study we focussed on a single initial starting population structure to ascertain how different stage structures can come about from the same average starting point. Dynamics will change if stage-structures vary [78,79], future work could apply more localised stage structures where known, especially if modelling local interventions.

Our model was developed with the UK cat population in mind and caution must be used when extrapolating predictions or applying the model to other geographies which may have different connectivity, capacities, density-dependent mechanisms, vital rates and stage structures. Instead, the model serves as a framework, which should be refined and updated in a way that best captures the locality of interest.

Additional studies of the demography of the feral and stray cat subpopulations are particularly necessary. With no UK studies available for the unowned-cat parameters, this study relied on data collected in other international contexts and attempted to account for uncertainty by incorporating a range of variation in these parameters. However, the true variation and demographics may in fact be different to what this study has assumed. Since, free-roaming unowned cats are often the primary cause for concern, more robust estimators of their demographics across different UK environments are needed and this will require more focussed studies, such as longitudinal research systematically monitoring populations through time, to provide detailed individual life history data. Such research could also look into density-dependent processes. Within the free-roaming unowned cat population we may expect lower survival and/or fecundity with increased numbers of cats due to increased competition for resources and/or increased infectious disease, however, there is currently too little data to parameterise this process. Another important area is the influence of perturbation to the stage structure on population dynamics, such as through cat importation, with the proportion of owned cats coming from abroad recently recorded as 5% in 2022 [2,55].

Additionally, our model does not account for covariation in demographic traits. Trade-offs are expected to occur among competing demographic traits, for example resource limitation prevents organisms from simultaneously maximising their survival and reproductive output. Such trade-offs have been found to be important influencers of population dynamics in other mammals [80]. We are currently lacking empirical insight into the nature of these trade-offs in cats, however, food and the availability of other resources are critical factors determining fitness and will limit investment in both survival and reproduction. Therefore, whilst covariation may occur in all subpopulations it may be particularly important in free-roaming unowned cats that are more reliant on external environmental factors and associated resource availability. In addition to covariation, there may be occurrences where interventions have an indirect impact on vital rates and/or transitions that are not the target. For example, it has been suggested that targeted neutering of the owned cat subpopulation may select for kittens that are less suitable for socialisation to people [81] (which could have implications for rehoming transitions) or targeted neutering of specific subgroups of owned cats such as non-pedigrees may select for kittens with differing reproductive or survival rates [82], or health or behavioural needs [83], potentially impacting both vital rates and/or rehoming transitions.

Given further increases in data availability and quality as described above, this work will provide a novel testbed to explore the influence of naturally occurring processes (e.g. disease outbreaks) and a range of competing interventions, such as those that impact reproduction (e.g. neutering and supplemental feeding), influence movement between subpopulations (e.g. reduced straying or increased rehoming from shelters) and those that affect survival (e.g. changes to care provision). Additionally, our monthly time-step provides a means to explore the timing of any interventions, especially given the seasonality of reproduction.

### Conclusion

Our conclusions are necessarily limited by available demographic data, especially for feral and stray populations where data is sparse within the UK. Consequently we account for this uncertainty within the modelling approach, but further studies to obtain UK-based estimates would further aid to refine this model and reduce the uncertainty of its findings. As more data become available, we expect the model’s predictions to become more realistic. The main advantage of this model is that it provides a framework that can be easily modified for implementation in different geographical areas, to model any of the cat subpopulations or updated with new information as it comes to light. Our scenarios demonstrate a mechanism by which owned-cat neutering practices can profoundly influence the dynamics of all cat subpopulations. By using owned-cat neutering as our example interventions, this study highlights more generally the importance of understanding and modelling the links between domestic cat subpopulations, to improve the accuracy of modelled outcomes and better inform management strategies.

## Supporting information

S1 Fig (a) The absolute numbers of cats that reside in each subpopulation (shelter cats are not as visible due to the low proportion and that reside in this subpopulation and the (b) percentages of cats in the unowned- stray, feral and shelter subpopulations following ten-year projections of populations with low (90%), medium (95%) and high (98%) proportions of the adult cat population neutered, Each simulation assumed a starting abundance of 100,000 cats (indicated by the line).
(DOCX)

S2 Fig (a-c) The median absolute number of cats that reside in each in each subpopulation (shelter cats are not as visible due to the low proportion and that reside in this subpopulation) and the (d-f) median percentages of cats in the unowned- stray, feral and shelter subpopulations following ten-year projections of populations with low (90%), medium (95%) and high (98%) proportions of the adult cat population neutered, under scenarios where between 5% and 50% of the owned cats are neutered prepubertally to prevent parturition at 6 months. Each simulation assumed a starting abundance of 100,000 cats (indicated by the line).
(DOCX)

S1 Table Underlying vital rates used to parameterise the cat population model. Probabilities were converted into an approximate monthly rate using the formula p~1-(1-p)^1/n^, where n = the number of months the original parameter was recorded over. Where data were unavailable, informed estimates based on the expected behaviour of the system were included as indicated below. Parameters that vary due to density dependent processes [DD] and vary per scenarios 1 [S1] and 2 [S2] are also included.
(DOCX)

S2 Table Transition matrix (matU) underlying matrix structure and vital rates. (XLSX)

S3 Table Fertility matrix (matF) underlying matrix structure and vital rates. (XLSX)
