# Supplementary material for: Cat: Empirical modelling of Felis catus population dynamics in the UK
Source: PLoS One. 2023 Jul 12;18(7):e0287841. doi: 10.1371/journal.pone.0287841 (PMC10337951; doi:10.1371/journal.pone.0287841)
Supplement: S1 Fig — (a) The absolute numbers of cats that reside in each subpopulation (shelter cats are not as visible due to the low proportion and that reside in this subpopulation and the (b) percentages of cats in the unowned- stray, feral and shelter subpopulations following ten-year projections of populations with low (90%), medium (95%) and high (98%) proportions of the adult cat population neutered, Each simulation assumed a starting abundance of 100,000 cats (indicated by the line). (DOCX) [file pone.0287841.s001.docx]

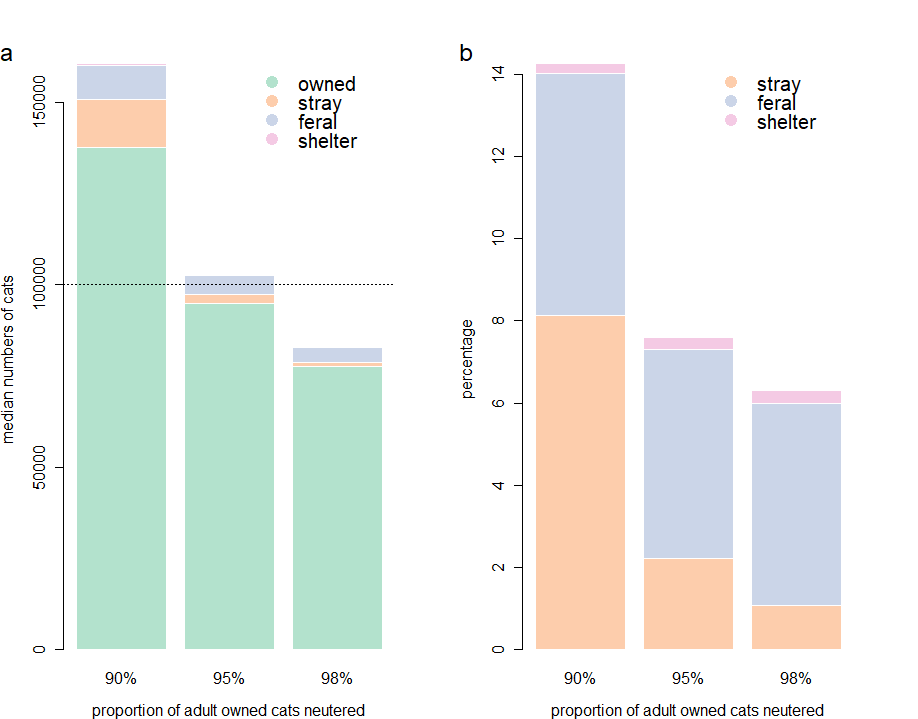


Figure S1. (a) The absolute numbers of cats that reside in each subpopulation. Shelter cats are not as visible due to the low proportion and that reside in this subpopulation and the (b) percentages of cats in the unowned- stray, feral and shelter subpopulations following ten-year projections of populations with low (90%), medium (95%) and high (98%) proportions of the adult cat population neutered, Each simulation assumed a starting abundance of 100,000 cats (indicated by the line).
