# Supplementary material for: Cat: Empirical modelling of Felis catus population dynamics in the UK
Source: PLoS One. 2023 Jul 12;18(7):e0287841. doi: 10.1371/journal.pone.0287841 (PMC10337951; doi:10.1371/journal.pone.0287841)
Supplement: S2 Fig — (a-c) The median absolute number of cats that reside in each in each subpopulation (shelter cats are not as visible due to the low proportion and that reside in this subpopulation) and the (d-f) median percentages of cats in the unowned- stray, feral and shelter subpopulations following ten-year projections of populations with low (90%), medium (95%) and high (98%) proportions of the adult cat population neutered, under scenarios where between 5% and 50% of the owned cats are neutered prepubertally to prevent parturition at 6 months. Each simulation assumed a starting abundance of 100,000 cats (indicated by the line). (DOCX) [file pone.0287841.s002.docx]

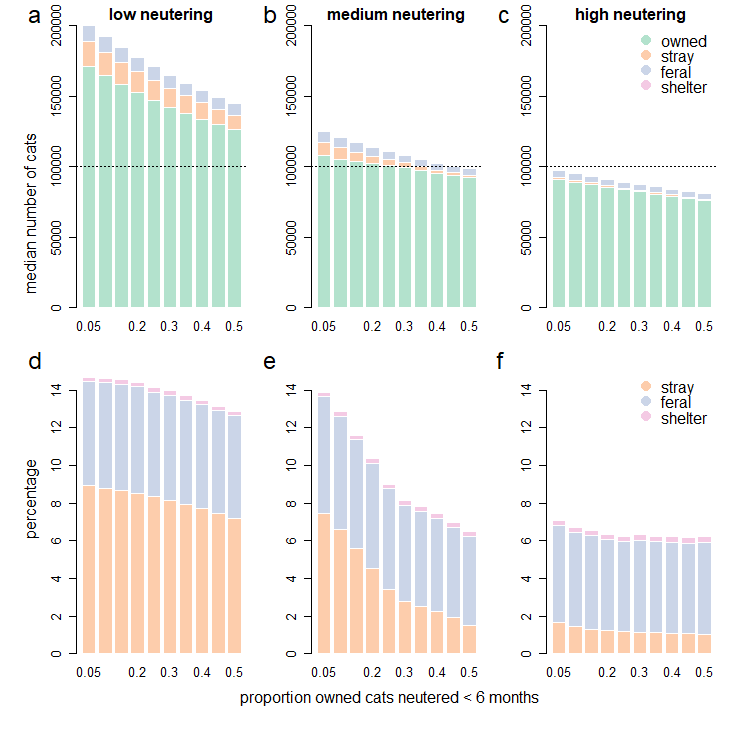


Figure S2. (a-c) The median absolute number of cats that reside in each in each subpopulation (shelter cats are not as visible due to the low proportion and that reside in this subpopulation ) and the (d-f) median percentages of cats in the unowned- stray, feral and shelter subpopulations following ten-year projections of populations with low (90%), medium (95%) and high (98%) proportions of the adult cat population neutered, under scenarios where between 5% and 50% of the owned cats are neutered prepubertally to prevent parturition at 6 months. Each simulation assumed a starting abundance of 100,000 cats (indicated by the line).
