# Supplementary material for: Cat: Empirical modelling of Felis catus population dynamics in the UK
Source: PLoS One. 2023 Jul 12;18(7):e0287841. doi: 10.1371/journal.pone.0287841 (PMC10337951; doi:10.1371/journal.pone.0287841)
Supplement: S1 Table — Probabilities were converted into an approximate monthly rate using the formula p~1-(1-p)1/n, where n = the number of months the original parameter was recorded over. Where data were unavailable, informed estimates based on the expected behaviour of the system were included as indicated below. Parameters that vary due to density dependent processes [DD] and vary per scenarios 1 [S1] and 2 [S2] are also included. (DOCX) [file pone.0287841.s003.docx]

**Supplementary Table 1.** Underlying female-specific vital rates used to parameterise the cat population model. Probabilities were converted into an approximate monthly rate using the formula p~1-(1-p)^1/n^, where n=the number of months the original parameter was recorded over. Where data were unavailable, informed estimates based on the expected behaviour of the system were included as indicated below. Parameters that vary due to density dependent processes [DD] and vary per scenarios 1 [S1] and 2 [S2] are also included.

| Parameter | Description | Monthly value (variance when appropriate) [other values used due to density dependent processes or scenario testing] | Derivation |
| --- | --- | --- | --- |
| **Feral cats** |  |  |  |
| φFK | Survival of a feral kitten | 0.81 (0.001)  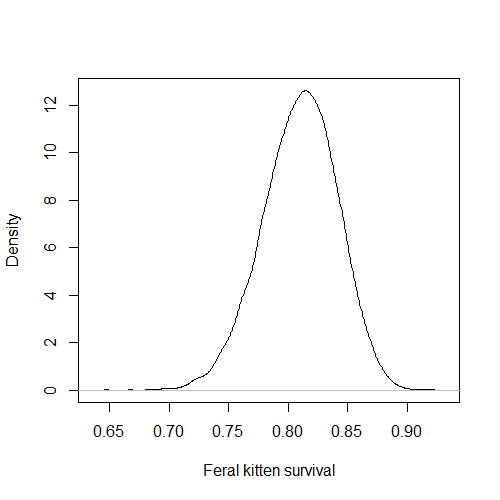 | No UK or urban studies, consequently taken from international data (USA, Israel, France, Australia), with monthly rates ranging from 0.707 to 0.915 depending on whether cats have caretakers, and geographic region [1–5]. |
| φ FJ | Survival of a feral juvenile | 0.92 (0.0005)  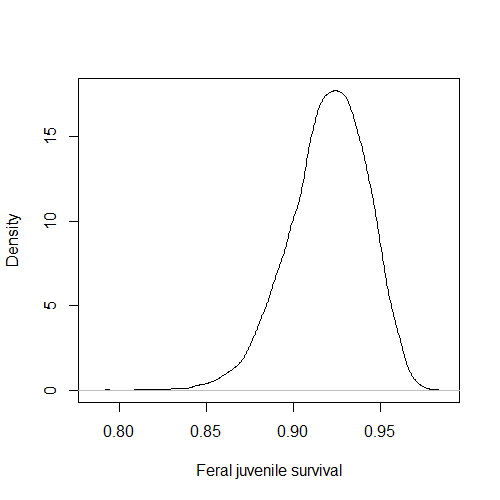 | No UK studies, taken from international studies (USA France) ranged from 0.86 to 0.96 [2,6]. |
| φ FA | Survival of a feral adult | 0.96 (0.0001)  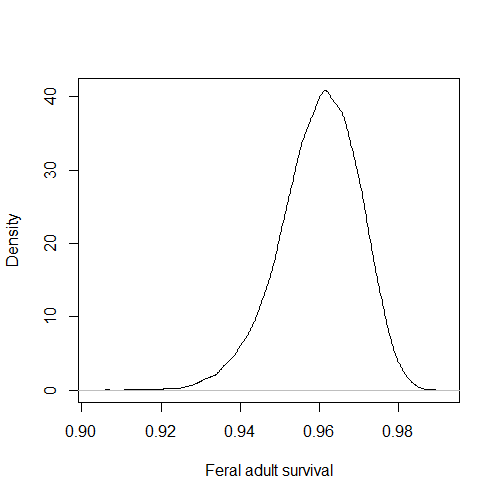 | No UK studies, taken average from international studies (USA, France, Hawaii, Australia) values range from 0.93 to 0.99 [1,3,6–9] |
| φ FS | Survival of a feral senior | 0.9 (0.0003)  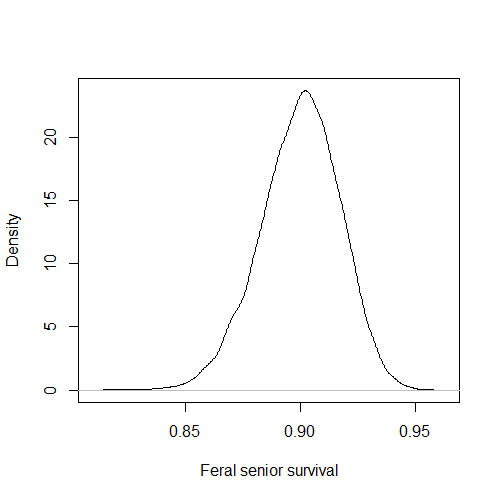 | No UK Studies, feral cats are not thought to survive this long, average lifespan 4-5 years, in a French study [9] and only 1% reported to survive beyond 7 years in Australian review [1] , so set survival low. |
| ωFtoSh | Transition of feral to shelter^1^ | 0.003 [DD; 0.00075, 0.00225] | Model derived.  No information available calculated based on an assumption on the number of feral kittens in the UK and the number of feral kittens entering shelters. Between 0.2 and 0.5% of feral kitten population. Estimate used and density-dependent values model derived. |
| ωFtoO | Transition of feral to owned^1^ | 0.02 [DD; 0.0002, 0.005] | Model derived.  No information available calculated based on an assumption on the number of feral kittens in the UK and the number of feral kittens entering homes from industry data. Estimate used and density-dependent values model derived. |
| ωFUtoFN | Transition feral unneutered to feral neutered^2^ | 0.01 | Limited information, based on little to no care received in some areas [10] and limited neutering generally as per previous international models [11] |
| ψFJ | birth rate of a feral juvenile | 1.5 x Monthly seasonal probability^3^ | No UK studies or data on juveniles, assume average litter size to be 3 with 1 litter occurring when cats < 1 year of age [12] |
| ψFA | birth rate of a feral adult | 2.5 x Monthly seasonal probability^3^ | No UK studies, data taken from USA and Australia. Based on average litter size of 3.5 with published ranges between 3 and 4[2,4,13–15] and 1.4 litters per year [3,4] |
| **Shelter cats** |  |  |  |
| φShK | Survival of a shelter kitten | 0.974 (0.00001)  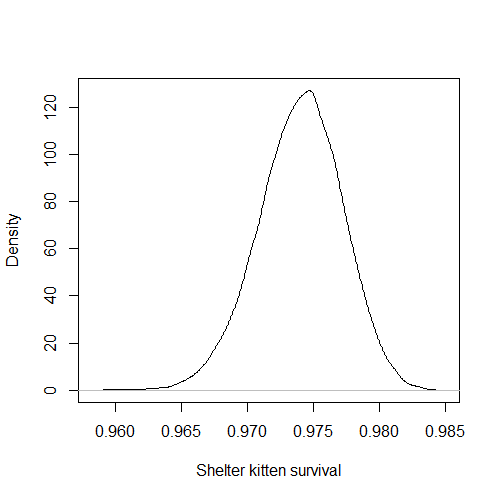 | Average age-specific value from unpublished estimates (CP 2022), overall average proportion of cats that do not die in care (0.96) corresponds with published estimates range from 0.87-0.96 published [16–18]. Whilst, UK studies of age-specific mortality is unpublished, it has been previously found that kittens and older cats have the increased risk of mortality [17]. |
| φShJ | Survival of a shelter juvenile | 0.993 (0.00001)  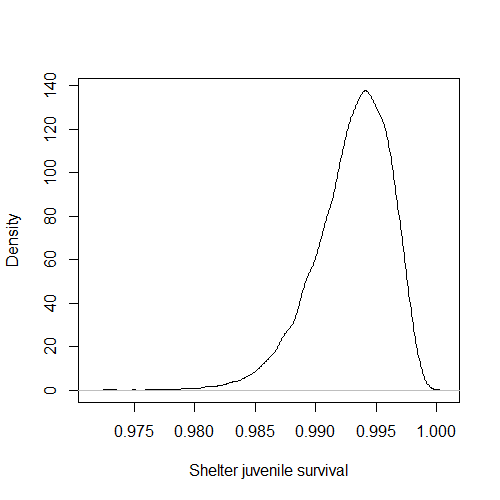 | As above |
| φShA | Survival of a shelter adult | 0.985 (0.00001)  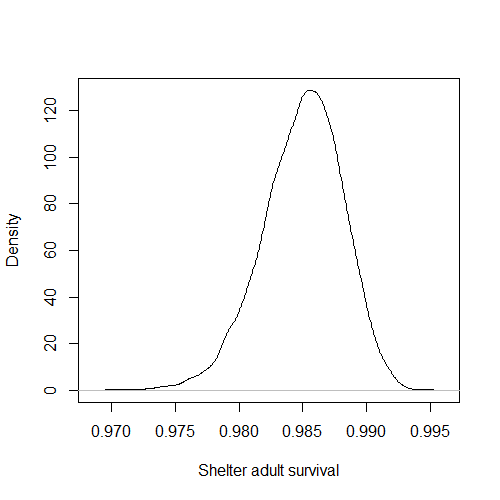 | As above |
| φShS | Survival of a shelter senior | 0.9 (0.00001)  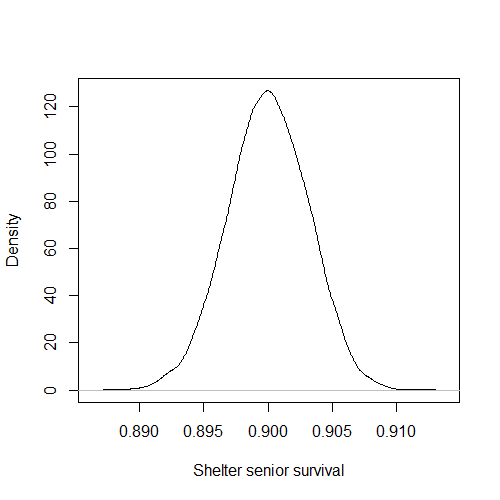 | As above |
| ωShtoO | Transition of shelter to owned | 0.63 | The median length of stay in a shelter was approximated to be 45 days based on unpublished UK figures and similar to published estimates [19,20]. |
| **Stray cats** |  |  |  |
| φStK | Survival of a stray kitten | 0.918 (0.00003)  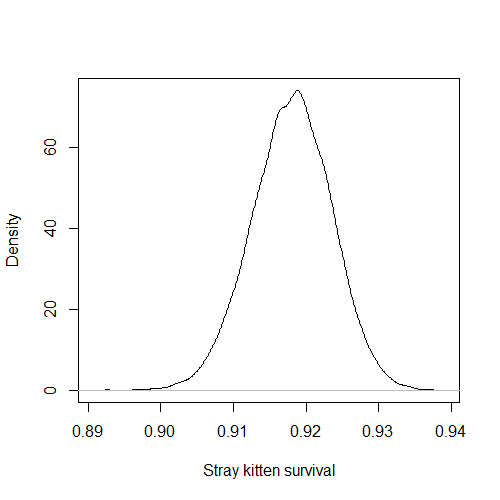 | No UK studies consequently similar value used to international data (USA, Israel), 0.91-0.92 [2,5] |
| φStJ | Survival of a stray juvenile | 0.97 (0.00003)  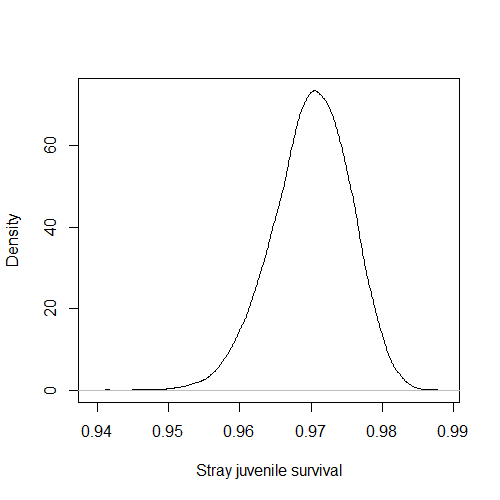 | No information, taken from adult stray figures below |
| φStA | Survival of a stray adult | 0.97 (0.00003)  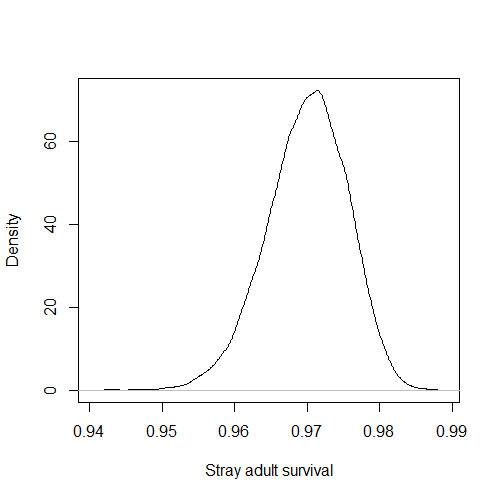 | No UK studies, taken from USA studies, which spanned 0.948-0.988 [3,15,21] |
| φStS | Survival of a stray senior | 0.9 (0.00003)  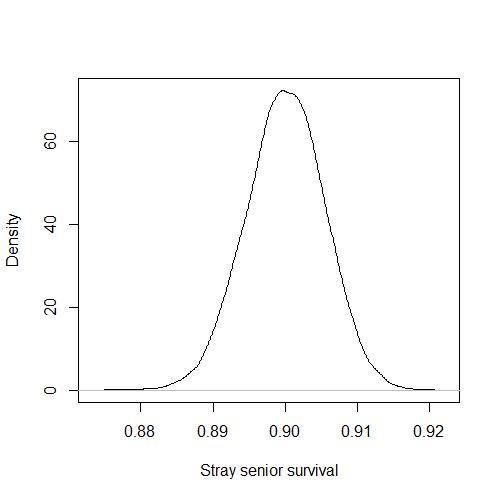 | No information, use lower survival rate similar to feral cats. |
| ωSttoF | Transition of stray to feral^4^ | 0.14186 | Model derived: calculated as the likelihood of not going into a shelter or becoming owned whilst a kitten. |
| ωSttoO | Transition of stray to owned | 0.04 [DD; 0.0004, 0.018] | Model derived.  No information available calculated based on an assumption on the number of stray cats in the UK and the number of strays entering homes from industry data. Estimate used and density-dependent values model derived. |
| ωSttoSh | Transitions of stray to shelter | 0.03 [DD;0.0075, 0.0225] | Model derived.  No information available calculated based on an assumption on the number of stray cats in the UK and the number of strays entering homes from industry data. Estimate used and density-dependent values model derived. |
| ωStUtoOKN | Transition unneutered to neutered upon rehoming when transitioning from a kitten to a juvenile | 0.41  [S2; 0.05, 0.1, 0.15, 0.2, 0.25, 0.3, 0.35, 0.4, 0.45, 0.5] | Use owned data assuming neuter rates of cats entering owned population follow owned population rates |
| ωStUtoOJN | Transition unneutered to neutered upon rehoming when a juvenile | 0.61  [S2; Low neutering; 0.37442215, 0.4073473, 0.44027245, 0.4731976, 0.50612275, 0.5390479, 0.57197305, 0.6048982, 0.63782335, 0.6707485  Medium neutering; 0.37442215, 0.4073473, 0.44027245, 0.4731976, 0.50612275, 0.5390479, 0.57197305, 0.6048982, 0.63782335, 0.6707485  High neutering; 0.37442215, 0.4073473, 0.44027245, 0.4731976, 0.50612275, 0.5390479, 0.57197305, 0.6048982, 0.63782335, 0.6707485] | Use owned data assuming neuter rates of cats entering owned population follow owned population rates |
| ωStAUtoOAN | Transition unneutered to neutered upon rehoming when an adult | 0.95 [S1 0.90, 0.95, 0.98]  [S2; Low neutering; 0.90  Medium neutering; 0.95  High neutering; 0.98] | Use owned data assuming neuter rates of cats entering owned population follow owned population rates |
| ψStJ^6^ | birth rate of a stray juvenile | 1.5 x Monthly seasonal probability^3^ | No published studies, see feral cats for derivation |
| ψStA^6^ | birth rate of a stray adult | 2.5 x Monthly seasonal probability^3^ | No published studies, see feral cats for derivation |
| **Owned cats** |  |  |  |
| φOK | Survival of an owned kitten | 0.97 (0.000001)  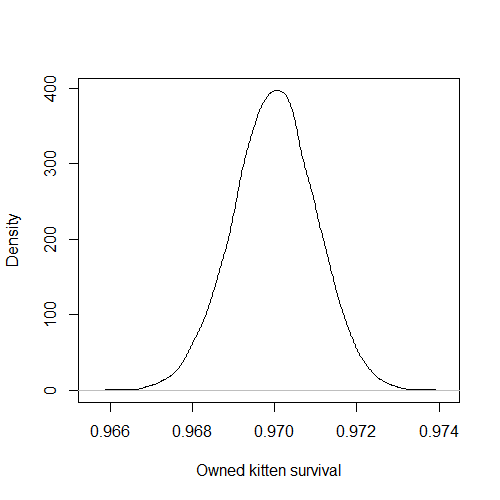 | Based on unpublished data on outcomes for kittens born in the past 12 months from a UK public survey [22], similar to published estimates [23] |
| φOJ | Survival of an owned juvenile | 0.995 (0.000001)  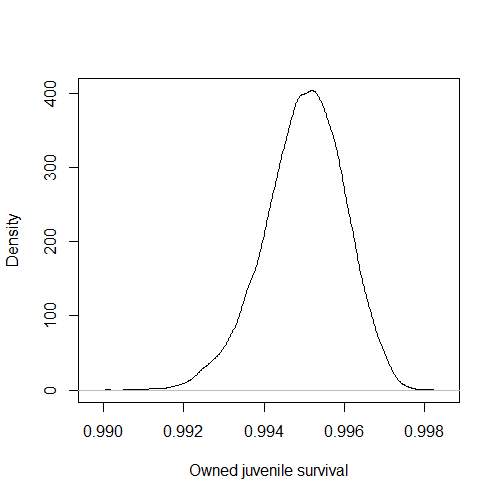 | No specific estimates for juveniles within the UK. Survival estimates when accounting for mortality due to RTAs alone are approximately 0.996 [24], therefore applied adult estimate to account for other causes of mortality |
| φOA | Survival of an owned adult | 0.995 (0.000001)  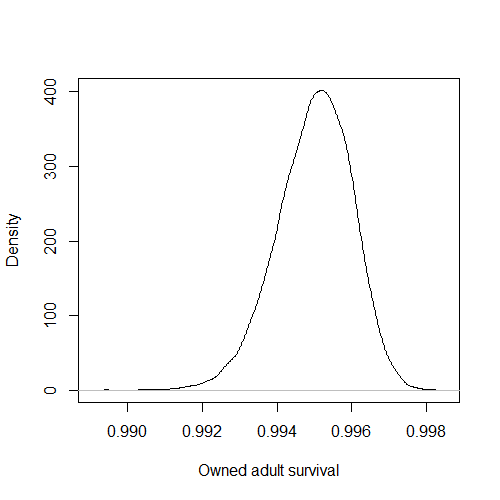 | Unpublished data on outcomes for cats in the past 12 months from a UK public survey [22], aligned with a UK veterinary surveillance study calculated from [25] and similar to international estimates 0.9947-0.9957[21,26] |
| φOS | Survival of an owned senior | 0.98 (0.000001)  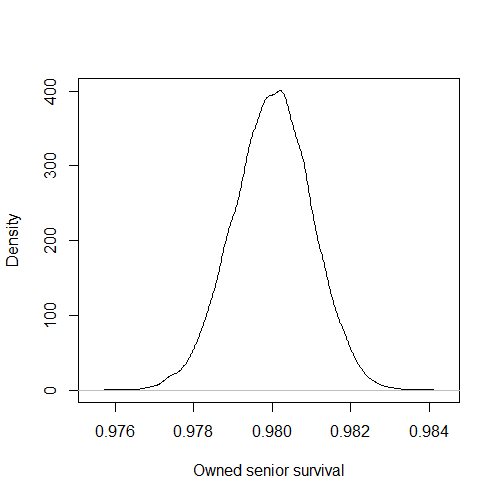 | Estimated so <1% of cats reach 26 years per [25] |
| ωOtoSh | Transition from owned to shelter ^5^ | 0.002 [DD 0.001, 0.0015] | Limited information approximated using 3 approaches (1) proportion of owned cats in shelter intake (CP) and estimates of cats in shelter from Cat Chat and average cats in shelter [18](0.0017); (2) calculated from unpublished data collected as part of [22]  (0.0019) and (3) model derived to create an adequate test-bed |
| ωOtoSt | Transition from owned to stray ^5^ | 0.0009 [DD 0.0018, 0.001125] | Limited information approximated using 3 approaches (1) proportion of owned cats in shelter intake from unpublished data (0.00085); (2) calculated from unpublished data collected as part of [22]  (0.00093) and (3) model derived to create an adequate test-bed |
| ωOKUtoOJN | Transition owned kitten unneutered to owned juvenile neutered | 0.41  [S2; 0.05, 0.1, 0.15, 0.2, 0.25, 0.3, 0.35, 0.4, 0.45, 0.5] | CP Cat Owner survey 2022, [27] (proportion neutered by 6-months) |
| ωOJUtoOJN | Transition owned juvenile unneutered to juvenile neutered | 0.13 | Monthly neutering rate estimated to obtain proportion neutered at 12 months as described in CP Cat Owner survey and [27] |
| ωOAUtoOAN | Transition owned adult unneutered to owned adult neutered at adult stage | 0.805 [S1; 0.61, 0.805, 0.92]  [S2; Low neutering; 0.757249083, 0.743762921, 0.728690152, 0.711733286, 0.692515505, 0.670552327, 0.645210198, 0.615644382, 0.580702962,0.538773258;  Medium neutering; 0.878624542, 0.871881461, 0.864345076, 0.855866643, 0.846257753, 0.835276164,  0.822605099, 0.807822191, 0.790351481, 0.769386629  High neutering; 0.951449817, 0.948752584, 0.94573803, 0.942346657, 0.938503101, 0.934110465, 0.92904204,0.923128876,  0.916140592, 0.907754652] | Calculated as a fixed rate upon entering adulthood to obtain the proportion of cats neutered [10,27–29] |
| ψOJ^6^ | birth rate of an owned juvenile | 1.4 x Monthly seasonal probability^3^ | As below but assume reproduced reproductive output due to later onset of breeding as although cats can become pregnant earlier, on average this occurs after six months [30] |
| ψOA^6^ | birth rate of an owned adult | 2.1 x Monthly seasonal probability^3^ | 1.5 average number of litters a female cat has past 12 months (unpublished, collected as part of [22]), aligns with estimates of published estimates, which state on average 1 or 2 litters per year [3,31] and an average litter size of 4 kittens [3,23,28] Also account for approximately 70% of unneutered cats having outdoor access and the potential to breed [32,33] and seasonality in reproduction [34] as described in main manuscript. |
| Ageing- Stationary age within stage structure for a given subpopulation (SP) |  |  |  |
| ωKtoJ | Kitten to Juvenile | $\frac{\varphi_{SP,K}^{5}}{\Sigma_{j=0}^{6}\varphi_{SP,K}^{j}}$ | [35] |
| ωJtoA | Juvenile to Adult | $\frac{\varphi_{SP,J}^{5}}{\Sigma_{j=0}^{6}\varphi_{SP,J}^{j}}$ | [35] |
| ωAtoS | Adult to Senior | $\frac{\varphi_{SP,A}^{119}}{\Sigma_{j=0}^{120}\varphi_{SP,A}^{j}}$ | [35] |
| **Starting population sizes**  **(assumes proportion female are consistent across all subpopulations)** |  |  |  |
| Feral n_1,_ | Starting population size feral cats, given a total starting population of 100,000 | 5206.4 | 5.2% population feral  Assumes a total UK population of 621,000 feral cats out of a total population of 11,927,972 cats.  Calculation based on an assumption of 900,000 unowned cats comprising urban, rural and farm cats[36,37]. Assumes 69% are feral as found from unpublished field data from community engagement programs (e.g. [38]) and similar to other studies that found between 66% and 75% of unowned cats to be feral, rather than socialised strays [3,39] |
| Shelter n_1_ | Starting population size shelter cats, given a total starting population of 100,000 | 232.1 | 0.2% population shelter  Assumes a total UK population of 27,702 cats residing in shelters at any one time out of a total population of 11,927,972 cats. Calculated based on an estimate of 1,026 shelters in the UK [40] with a median number of cats housed 27 [41] |
| Stray n_1_ | Starting population size stray cats, given a total starting population of 100,000 | 2339.2 | 2.3% population stray 279,000 stray cats out of a total population of 11,927,972 cats  Number of unowned cats estimated as described for feral cats above, assuming 31% are stray |
| Ownedl n_1_ | Starting population size owned cats, given a total starting population of 100,000 | 92, 222.3 | 92.2% population owned  11,000,000 pet cats in the UK [22] out of a total population of 11,927,972 cats |

^1^Only applied to the kitten stage, assuming the only kittens pre-socialisation would be taken into owned homes or shelters

^2^Can be applied from juvenile or adult or both– currently applied to adult due to limited data available

^3^ Seasonal reproduction probability vector (0.0366667,0.0366667, 0.0366667, 0.1300000, 0.1300000, 0.1300000, 0.1300000, 0.1300000, 0.1300000, 0.0366667, 0.0366667, 0.0366667)

^4^Only applied to kittens assuming a lack of socialisation whilst straying

^5^ Model framework allows age specific transitions for flexibility in modelling approach, fixed rate modelled for all categories currently

^6^ Already pregnant shelter cats were assumed to give birth after arrival at the shelter during the first month, therefore had the fecundity of their origin subpopulation. However, the model assumes a reduced birth rate with earlier stage pregnancies terminated via pregnant spays [30,42]

**References**

1. Denny EA, Dickman CR. Review of cat ecology and management strategies in Australia: A report for the invasive animals cooperative research centre. 2010.

2. Schmidt PM, Lopez RR, Collier BA. Survival, Fecundity, and Movements of Free-Roaming Cats. Journal of Wildlife Management. 2007;71: 915–919. doi:10.2193/2006-066

3. Kass PH, Johnson KL, Weng HY. Evaluation of animal control measures on pet demographics in Santa Clara County, California, 1993-2006. PeerJ. 2013;2013: 1993–2006. doi:10.7717/peerj.18

4. Nutter FB, Levine JF, Stoskopf MK. Free-roaming cat survival rate. Journal of American Veterinary Medical Association. 2004;225: 1399–1402.

5. Gunther I, Finkler H, Terkel J. Demographic differences between urban feeding groups of neutered and sexually intact free-roaming cats following a trap-neuter-return procedure. JAVMA. 2011;238.

6. Devillard S, Say L, Pontier D. Dispersal pattern of domestic cats (Felis catus) in a promiscuous urban population: Do females disperse or die? Journal of Animal Ecology. 2003;72: 203–211. doi:10.1046/j.1365-2656.2003.00692.x

7. Gehrt SD, Wilson EC, Brown JL, Anchor C. Population Ecology of Free-Roaming Cats and Interference Competition by Coyotes in Urban Parks. PLoS One. 2013;8: 1–11. doi:10.1371/journal.pone.0075718

8. Danner RM, Farmer C, Hess SC, Stephens RM, Banko PC. Survival of Feral Cats, Felis catus (Carnivora: Felidae), on Mauna Kea, Hawai’i, Based on Tooth Cementum Lines . Pac Sci. 2010;64: 381–389. doi:10.2984/64.3.381

9. Kaeuffer R, Pontier D, Devillard S, Perrin N, Kaeuffer R. Effective size of two feral domestic cat populations (Felis catus L.): Effect of the mating system. Mol Ecol. 2004;13: 483–490. doi:10.1046/j.1365-294X.2003.02046.x

10. Roberts C, Gruffydd-Jones TJ, Clements J, Jones TW, Farnworth MJ, Murray JK. Cats on farms in the United Kingdom: numbers and preventive care. Veterinary Record. 2018;183: 23.

11. Flockhart DTT, Coe JB. Multistate matrix population model to assess the contributions and impacts on population abundance of domestic cats in urban areas including owned cats, unowned cats, and cats in shelters. PLoS ONE. 2018. doi:10.1371/journal.pone.0192139

12. Nutter FB, Levine JF, Stoskopf MK. Reproductive capacity of free-roaming domestic cats and kitten survival rate. J Am Vet Med Assoc. 2004;225: 1399–1402. doi:10.2460/javma.2004.225.1399

13. Molsher RL. Trapping and demographics of feral cats (Felis catus) in central New South Wales. Wildlife Research. 2001;28: 631–636. doi:10.1071/WR00027

14. Scott KC, Levy JK, Crawford PC. Characteristics of free-roaming cats evaluated in a trap-neuter-return program. J Am Vet Med Assoc. 2002;221: 1136–1138. doi:10.2460/javma.2002.221.1136

15. Ogan C V, Jurek RM. Biology and ecology of feral, free-roaming, and stray cats. Mesocarnivores of Northern California: biology, management, and survey techniques. 1998; 87–91.

16. Stavisky J. Too many cats: How owner beliefs contribute to overpopulation. Veterinary Record. 2014;174: 116–117. doi:10.1136/vr.g1100

17. Murray JK, Skillings E, Gruffydd-Jones TJ. A study of risk factors for cat mortality in adoption centres of a UK cat charity. J Feline Med Surg. 2008;10: 338–345. doi:10.1016/j.jfms.2008.01.005

18. Stavisky J, Brennan ML, Downes M, Dean R. Demographics and economic burden of un-owned cats and dogs in the UK: results of a 2010 census. BMC Vet Res. 2012;8: 163. Available: http://www.biomedcentral.com/1746-6148/8/163

19. Janke N, Berke O, Flockhart T, Bateman S, Coe JB. Risk factors affecting length of stay of cats in an animal shelter: A case study at the Guelph Humane Society, 2011–2016. Prev Vet Med. 2017;148: 44–48. doi:10.1016/j.prevetmed.2017.10.007

20. Miller H, Ward M, Beatty JA. Population characteristics of cats adopted from an urban cat shelter and the influence of physical traits and reason for surrender on length of stay. Animals. 2019;9. doi:10.3390/ani9110940

21. Horn JA, Mateus-Pinilla N, Warner RE, Heske EJ. Home range, habitat use, and activity patterns of free-roaming domestic cats. Journal of Wildlife Management. 2011;75: 1177–1185. doi:10.1002/jwmg.145

22. Cats Protection. Cats and Their Stats (CATS) UK 2022. 2022. Available: https://www.cats.org.uk/media/10005/cats-2021-full-report.pdf

23. Ström Holst B, Frössling J. The Swedish breeding cat: population description, infectious diseases and reproductive performance evaluated by a questionnaire. J Feline Med Surg. 2009;11: 793–802. doi:10.1016/j.jfms.2009.01.008

24. Wilson JL, Gruffydd-Jones TJ, Murray JK. Risk factors for road traffic accidents in cats up to age 12 months that were registered between 2010 and 2013 with the UK pet cat cohort ('Bristol Cats’). Veterinary Record. 2017;180: 195. doi:10.1136/vr.103859

25. O’Neill DG, Church DB, McGreevy PD, Thomson PC, Brodbelt DC. Longevity and mortality of cats attending primary care veterinary practices in England. J Feline Med Surg. 2015;17: 125–133. doi:10.1177/1098612X14536176

26. Lacheretz A, Moreau D, Cathelain H. Causes of death and life expectancy in carnivorous pets (I). Rev Med Vet (Toulouse). 2002;153: 819–822.

27. Sánchez-Vizcaíno F, Noble PJM, Jones PH, Menacere T, Buchan I, Reynolds S, et al. Demographics of dogs, cats, and rabbits attending veterinary practices in Great Britain as recorded in their electronic health records. BMC Vet Res. 2017;13: 1–13. doi:10.1186/s12917-017-1138-9

28. Bradshaw JWS, Horsfield GF, Allen JA, Robinson IH. Feral cats: Their role in the population dynamics of Felis catus. Appl Anim Behav Sci. 1999;65: 273–283. doi:10.1016/S0168-1591(99)00086-6

29. Murray JK, Roberts MA, Whitmarsh A, Gruffydd-Jones TJ. Survey of the characteristics of cats owned by households in the UK and factors affecting their neutered status. Veterinary Record. 2009;164: 137–141. doi:10.1136/vr.164.5.137

30. Joyce A, Yates D. Help stop teenage pregnancy!. Early-age neutering in cats. J Feline Med Surg. 2011;13: 3–10. doi:10.1016/j.jfms.2010.11.005

31. Welsh CP, Gruffydd-Jones TJ, Roberts MA, Murray JK. Poor owner knowledge of feline reproduction contributes to the high proportion of accidental litters born to UK pet cats. Veterinary Record. 2014;174: 118. doi:10.1136/vr.101909

32. PDSA. PDSA Animal wellbeing report (PAW) 2022. 2022. Available: https://www.pdsa.org.uk/what-we-do/pdsa-animal-wellbeing-report/paw-report-2022/pet-environment

33. Cats Protection. Cats and their stats report UK 2021. 2021. Available: https://www.cats.org.uk/media/10005/cats-2021-full-report.pdf

34. Jennett AL, Jennett NM, Hopping J, Yates D. Evidence for seasonal reproduction in UK domestic cats. J Feline Med Surg. 2016;18: 804–808. doi:10.1177/1098612X15595665

35. Kendall BE, Fujiwara M, Diaz-Lopez J, Schneider S, Voigt J, Wiesner S. Persistent problems in the construction of matrix population models. Ecol Modell. 2019;406: 33–43. doi:10.1016/j.ecolmodel.2019.03.011

36. McDonald JL, Skillings E. Human influences shape the first spatially explicit national estimate of urban unowned cat abundance. Sci Rep. 2021;11. doi:10.1038/s41598-021-99298-6

37. Roberts C, Gruffydd-Jones TJ, Clements J, Jones TW, Farnworth MJ, Murray JK. Cats on farms in the United Kingdom: Numbers and preventive care. Veterinary Record. British Veterinary Association; 2018. p. 23. doi:10.1136/vr.104746

38. McDonald JL, Farnworth MJ, Clements J. Integrating trap-neuter-return campaigns into a social framework: Developing long-term positive behavior change toward unowned cats in urban areas. Front Vet Sci. 2018;5. doi:10.3389/fvets.2018.00258

39. Levy JK, Gale DW, Gale LA. Evaluation of the effect of a long-term trap-neuter-return and adoption program on a free-roaming cat population. J Am Vet Med Assoc. 2003;222: 42–46. doi:10.2460/javma.2003.222.42

40. https://www.catchat.org. Cat Chat, The UK Cat Rescue Website.

41. Clark CCA, Gruffydd-Jones T, Murray JK. Number of cats and dogs in UK welfare organisations. Veterinary Record. 2012;170: 493. doi:10.1136/vr.100524

42. Dean R, Roberts M, Stavisky J. BSAVA manual of canine and feline shelter medicine. Principles of health and welfare in a multi-animal environment. British Small Animal Veterinary Association; 2018.
